# Repurposing T-type calcium channel blocker lomerizine as a therapeutic strategy for glioblastoma

**DOI:** 10.1172/jci.insight.182522

**Published:** 2026-03-24

**Authors:** Toshiya Ichinose, Sho Tamai, Nozomi Hirai, Takashi Maejima, Kosuke Nambu, Hemragul Sabit, Shingo Tanaka, Masashi Kinoshita, Masahiko Kobayashi, Michihiro Mieda, Atsushi Hirao, Mitsutoshi Nakada

**Affiliations:** 1 Department of Neurosurgery, Graduate School of Medical Science, Kanazawa University, Ishikawa, Japan.; 2 Department of Neurosurgery, Toho University Ohashi Medical Center, Tokyo, Japan.; 3 Department of Integrative Neurophysiology, Graduate School of Medical Sciences, and; 4 Division of Molecular Genetics, Cancer Research Institute, Kanazawa University, Ishikawa, Japan.

**Keywords:** Cell biology, Oncology, Brain cancer

## Abstract

Glioblastoma (GBM) is the most malignant primary brain tumor. The presence of glioma stem/initiating cells (GICs) is known to cause strong treatment resistance; therefore, GICs are a major target for GBM therapy, although there are no therapies targeting GICs clinically. To identify novel treatments for GBMs, we performed drug repurposing screening using GICs and identified the T-type calcium channel blocker lomerizine — a migraine prophylactic drug. Lomerizine inhibited proliferation, migration, invasion, and cell cycle progression and induced apoptosis in GICs and differentiated glioma cells. Lomerizine had antitumor effects by inactivating STAT3 in all cell lines. Furthermore, lomerizine also dephosphorylated AKT and ERK only in GICs and had strong tumor-suppressive ability. Lomerizine also reduced tumor volume and prolonged overall survival in vivo. Based on our data from in vitro and in vivo experiments, lomerizine has potential as a GBM therapeutic agent targeting both GICs and differentiated glioma cells and could benefit GBM patients.

## Abstract 2

T-type calcium channel blocker lomerizine has antitumor effects on both glioblastoma-stem and -differentiated cells, inhibiting proliferation, migration, invasion, and stemness.

## Graphical abstract

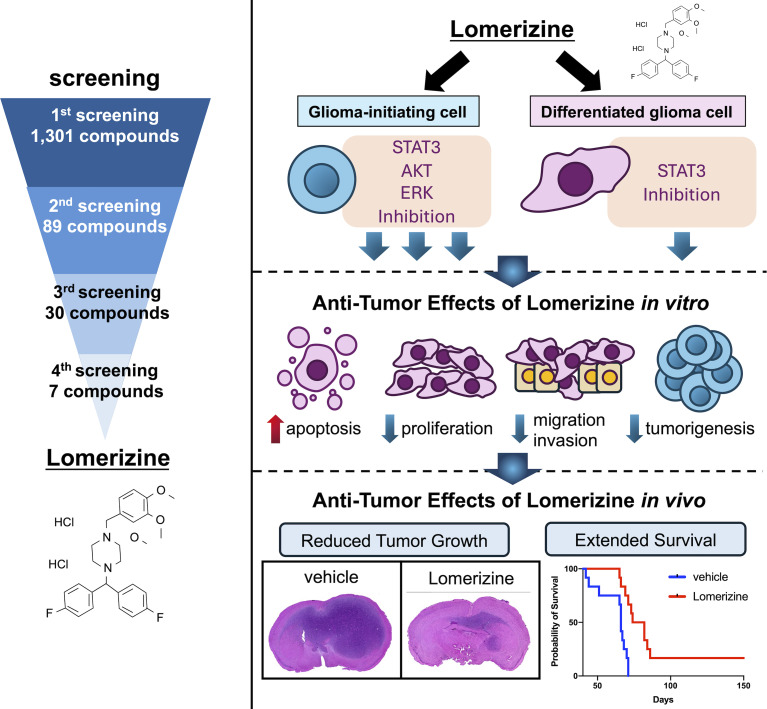

## Introduction

Glioblastoma (GBM), the most common primary intracranial tumor, is a highly aggressive, invasive, and fatal malignancy. Despite multimodal treatment, including maximal safe resection, radiotherapy, and chemotherapy, the prognosis remains poor, with an average survival duration of less than 2 years (1–3). Various factors lead to a lack of therapeutic success for GBM, including the rapid infiltration of tumor cells into the brain, inter- and intratumor heterogeneity, limited diffusion of therapeutic agents into the brain/tumor parenchyma owing to the presence of the blood-brain barrier, and strong resistance to radiation therapy and chemotherapy. The control and suppression of recurrent GBM are especially important to achieve long-term survival and ultimately cure patients. Resistance to chemotherapy and radiotherapy, as well as relapse, is attributed to a specific subpopulation of tumor cells with “stem-like” features, known as glioma stem/initiating cells (GICs) in GBM (4–6). Therefore, GICs may be a major target for GBM therapy, although there are currently no therapies that clinically target GICs.

Drug repurposing or repositioning is a therapeutic strategy used to identify new applications for previously approved or investigational drugs (7, 8). Repurposing is a more cost-effective and efficient approach to drug development as the safety profile of the drug is already established. Repurposing approved drugs can expedite the transition from clinical trials for new indications to clinical evaluation and approval, because approved drugs have already been extensively characterized with respect to their pharmacokinetic, pharmacodynamic, and toxicological properties (9). Examples include the use of thalidomide, originally a sleep drug, for multiple myeloma (10) and the osteoporosis drug raloxifene for invasive breast cancer (11). To identify novel treatments for glioma, we previously performed drug repurposing screening and identified several compounds with modest anti-glioma effects, primarily targeting GICs (12–16). However, none have yet been approved for clinical use.

In this study, we showed that the anti-migraine drug lomerizine, selected based on the results of drug screening against GICs, suppressed GBM cell proliferation, migration, and invasion in culture and tumor growth in a mouse model by influencing the STAT3, AKT, ERK, cell cycle regulatory kinase, and apoptosis effector signaling pathways. Lomerizine, an antagonist of T-type calcium channels, is a Food and Drug Administration–approved drug and is used as a first-line prophylactic drug for migraine in Japan (17). It exhibits excellent potential for glioma treatment, facilitated by its ability to cross the blood-brain barrier (BBB) owing to high lipid solubility (18, 19). Highly selective for the cerebral nervous system, it enables safe and sustained oral therapy to prevent nonfatal diseases without cardiovascular side effects such as hypoperfusion and tachycardia — a contrast observed with other calcium channel blockers (20). Despite being a highly desirable therapeutic agent for intracranial diseases, all previous reports on lomerizine in relation to the central nervous system have focused on its neuroprotective effects (21–24), with no reports on its anti-GBM effects. Our findings suggest that lomerizine is a promising agent for the treatment of GBM because of its ability to cross the BBB; additionally, it exerts anti-glioma effects on both GICs and differentiated glioma cells.

## Results

### Lomerizine inhibits glioma cell proliferation.

**Figure 1:**
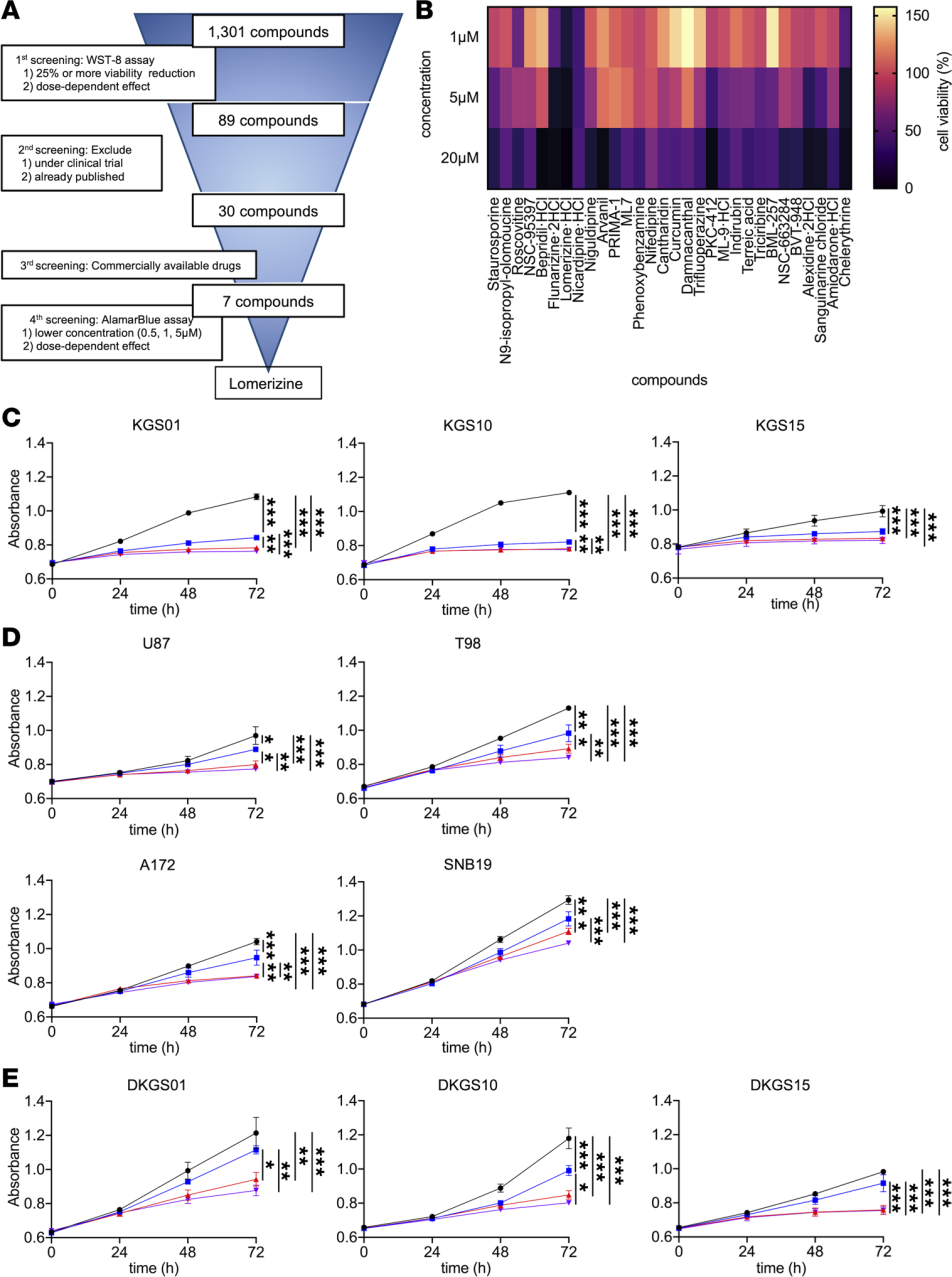
Drug screening and proliferation assay identified lomerizine as an effective agent with anti-GBM effects. (**A**) Drug screening scheme. A total of 1,301 compounds were gathered, and a 3-step procedure was performed to detect the candidate agents in this study. First, compounds that reduced the viability of GICs by at least 25% in a dose-dependent manner were selected. Second, we excluded compounds with previously reported effects on glioma and candidate compounds without regulatory approval. Finally, an Alamar blue assay was performed using two GICs (KGS01 and KGS10) with more detailed concentration distributions. (**B**) Heatmap of screened drugs that effectively inhibit GIC growth. All 1,301 compounds were investigated, and the 30 effective compounds that remained after second screening are shown in the heatmap. (**C**–**E**) Effect of lomerizine on GBM cell proliferation. Alamar blue proliferation assay was performed to demonstrate the effects of lomerizine treatment. Three different GICs (KGS01, KGS10, and KGS15) (**C**), 4 different glioma cell lines (U87, T98, A172, and SNB19) (**D**), and 3 differentiated GICs (DKGS01, DKGS10, and DKGS15) (**E**) were treated with lomerizine at 0 (DMSO), 1, and 5 μM, and each growth curve was analyzed. Plates were read using a microplate reader at 24, 48, and 72 hours. Results are shown as the mean ± SD of 6 independent experiments (**C**–**E**). Data were analyzed by 1-way ANOVA with Tukey’s multiple-comparison test. *P < 0.05, **P < 0.01, ***P < 0.005 vs. DMSO.

Of the 1,301 compounds, we identified 89 that inhibited the proliferation of GICs using the WST-8 cell proliferation assay (Figure 1A). Next, drugs that were undergoing clinical trials for GBM or that have already been reported to show anti-GBM effects were excluded, and 30 compounds were identified (Figure 1B). After the second screening of drug repurposing, 7 of 30 compounds (bepridil, flunarizine, nifedipine, nicardipine, niguldipine, amiodarone, and lomerizine) that showed antitumor effects against GIC were known to inhibit T-type calcium channels. Seven of the 30 compounds remaining after the second screening, including several T-type calcium blockers, are currently used clinically for diseases other than GBM and have not been reported to exert an influence on GBM. Among these, lomerizine was selected because of its strong antiproliferative effect on several GICs. We tested the antiproliferative efficacy of lomerizine against GICs and 4 common glioma cell lines. Lomerizine dose-dependently inhibited the proliferation of all 3 tested GICs — KGS01, KGS10, and KGS15 (Figure 1C). Interestingly, lomerizine also inhibited the proliferation of the 4 glioma cell lines in a concentration-dependent manner (Figure 1D). Next, we established differentiated GIC glioma cells (DKGS01, DKGS10, and DKGS15) and confirmed that lomerizine had an inhibitory effect on cell proliferation not only in GICs but also in differentiated cells (Figure 1E). The IC_50_ of lomerizine was lower in the GICs; therefore, they exhibited high sensitivity to lomerizine (Supplemental Figure 1; supplemental material available online with this article; https://doi.org/10.1172/jci.insight.182522DS1). These data suggest that lomerizine has a proliferation-inhibitory effect on both GICs and differentiated glioma cells and that a low dose of lomerizine has a stronger inhibitory effect on cell proliferation in GICs than in differentiated cells.

### Lomerizine induces apoptosis in glioma cells.

**Figure 2:**
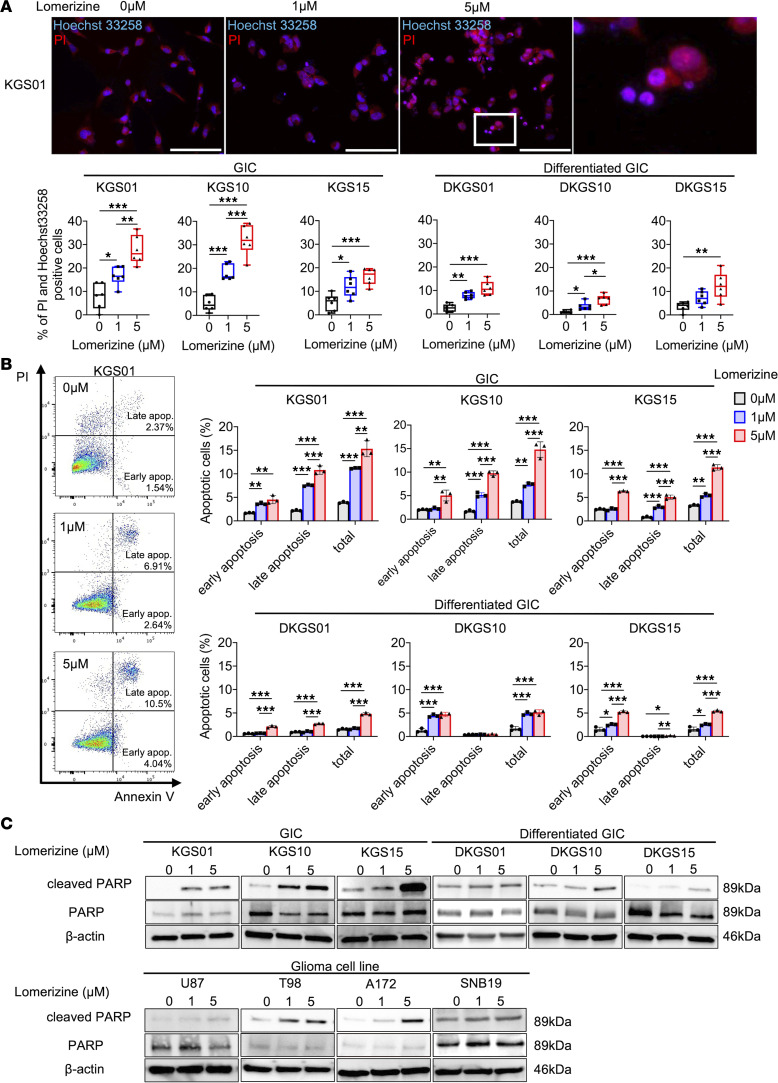
Lomerizine treatment induces apoptosis in glioma cell lines. The effect of lomerizine on the induction of apoptosis was evaluated using immunofluorescence, annexin V assays, and Western blotting in vitro. (**A**) Representative images of nuclear staining of KGS01 with or without lomerizine treatment for 24 hours, using Hoechst 33258 (blue) and propidium iodide (PI) (red). Apoptotic cells are indicated as condensed chromatin, with both Hoechst 33258– and PI-positive cells (pink). Inset shows higher magnification image (5×) of double-stained apoptotic cells. Bar graphs revealing the average number of double-stained apoptotic cells per high-power field for all GICs (KGS01, KGS10, and KGS15) and their differentiated cell lines (DKGS01, DKGS10, and DKGS15). (**B**) All GICs (KGS01, KGS10, and KGS15) and their differentiated cell lines (DKGS01, DKGS10, and DKGS15) were incubated with DMSO or lomerizine (1 μM or 5 μM) for 24 hours and then analyzed for apoptosis using annexin V/PI staining assays. Representative flow cytometry dot plots of apoptosis. The graphs represent the percentages of total apoptotic cells, including both the early and late apoptotic cells after treatment with different concentrations of lomerizine. Data are presented as the mean ± SEM of triplicate experiments. (**C**) Western blot analysis revealing the expression of cleaved PARP and PARP in all GICs and their differentiated cell lines treated with lomerizine for 24 hours. β-Actin was used as a loading control. Scale bars: 100 μm (**A**). Bars represent mean values ± SD (**A** and **B**). Data were analyzed by 1-way ANOVA with Tukey’s multiple-comparison test. *P < 0.05, **P < 0.01, ***P < 0.005 vs. DMSO.

We evaluated whether the inhibition of cell proliferation was associated with apoptosis induction and/or cell cycle distribution. First, we investigated whether lomerizine induces apoptosis in GBM cells. Indeed, lomerizine induced apoptosis in all cell lines, including GICs and differentiated glioma cells, as assessed by dual staining with antibodies and Hoechst 33258 and propidium iodide staining (Figure 2A and Supplemental Figure 2). Induction of apoptosis was particularly pronounced in GICs, which is consistent with the results of the proliferation assays. Lomerizine induced apoptosis in all cell lines, with the greatest potency against GICs, as evaluated by annexin V assays (Figure 2B and Supplemental Figure 3). In all glioma cell lines, the overall percentage of early or late apoptotic cells increased in a dose-dependent manner upon lomerizine treatment. In GICs, the percentage of late apoptotic cells was particularly high, indicating that apoptosis was induced early after drug exposure. Western blotting also revealed a dose-dependent increase in the expression of the apoptosis marker cleaved PARP in all glioma cell lines but was most potent in GICs, which is consistent with the results of immunofluorescence and annexin V assays (Figure 2C). In summary, lomerizine induced apoptosis in glioma cells. Induction of apoptosis was particularly pronounced in GICs, which strongly inhibited cell proliferation.

**Figure 3:**
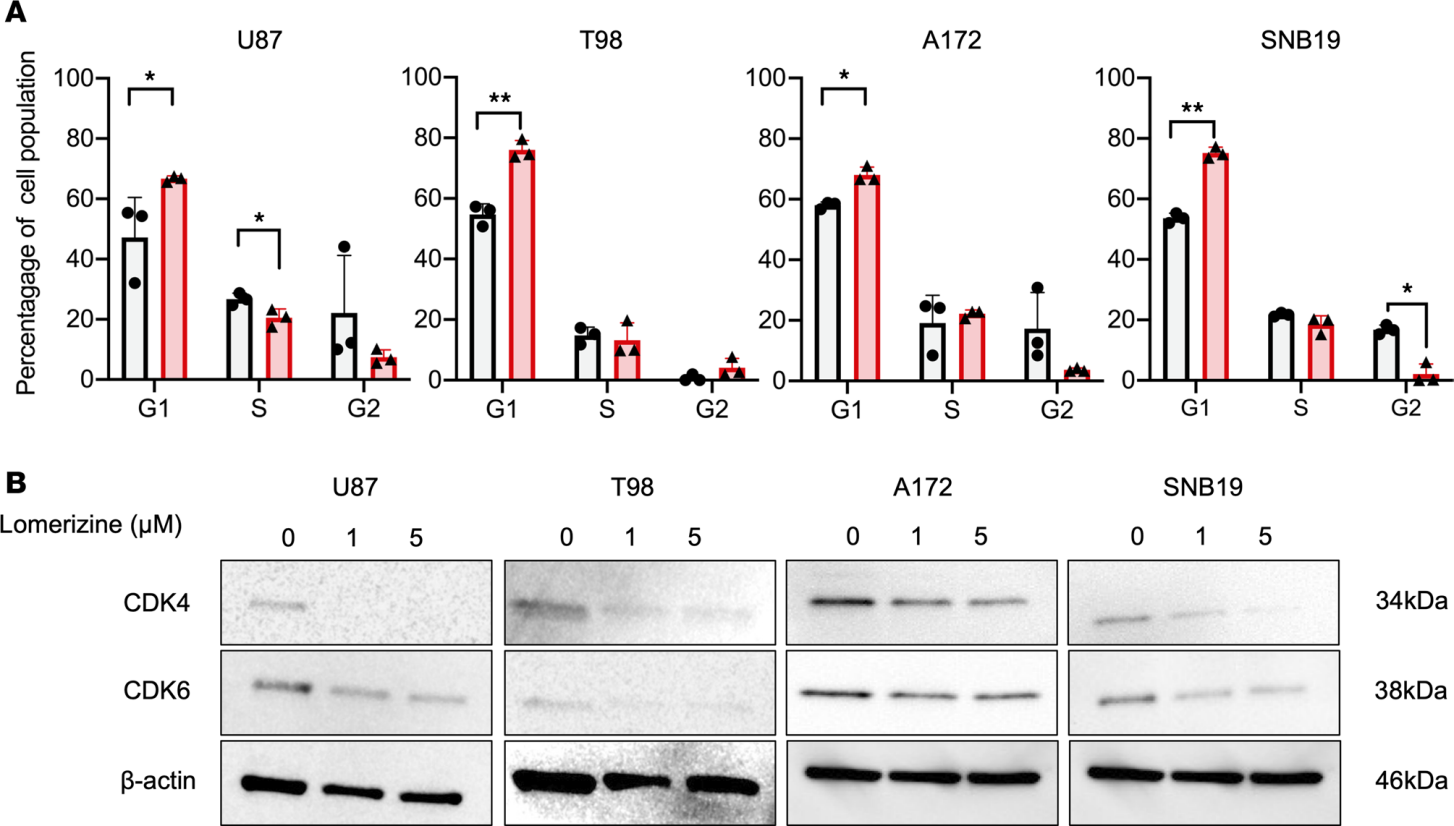
Effects of lomerizine on cell cycle of glioblastoma cells. (**A**) Four human glioma cell lines (U87, T98, A172, and SNB19) were incubated with DMSO or lomerizine (5 μM) for 24 hours, and then cell cycle analysis was performed using flow cytometry. The graphs represent the percentages of cells in G_0_/G_1_, S, and G_2_/M phases after treatment with/without lomerizine. Data are presented as the mean ± SEM of triplicate experiments. (**B**) Western blot analysis revealing the expression of cyclin-dependent kinase 4 (CDK4) and CDK6 in 4 human glioma cell lines treated with lomerizine for 24 hours. β-Actin was used as a loading control. Bars represent mean values ± SD (**A**). Data were analyzed by 2-tailed Student’s *t* test. *P < 0.05, **P < 0.01, ***P < 0.005 vs. DMSO.

Next, we examined the effects of lomerizine on the cell cycle distribution of glioma cells. Analysis by flow cytometry revealed that, based on the proportion of cell populations, the number of cells in the lomerizine-treated group was arrested in the G_1_ phase, indicating that cell proliferation was inhibited (Figure 3A). We examined the effects of lomerizine on cell cycle regulatory molecules by Western blotting. As a result, the expression of cyclin-dependent kinase 4 (CDK4) and CDK6, which subserves the cell cycle during the G_1_-to-S phase transition and proliferation, was suppressed by lomerizine in a dose-dependent manner in all human glioma lines (Figure 3B).

### Lomerizine inhibits glioma cell migration and invasion.

**Figure 4:**
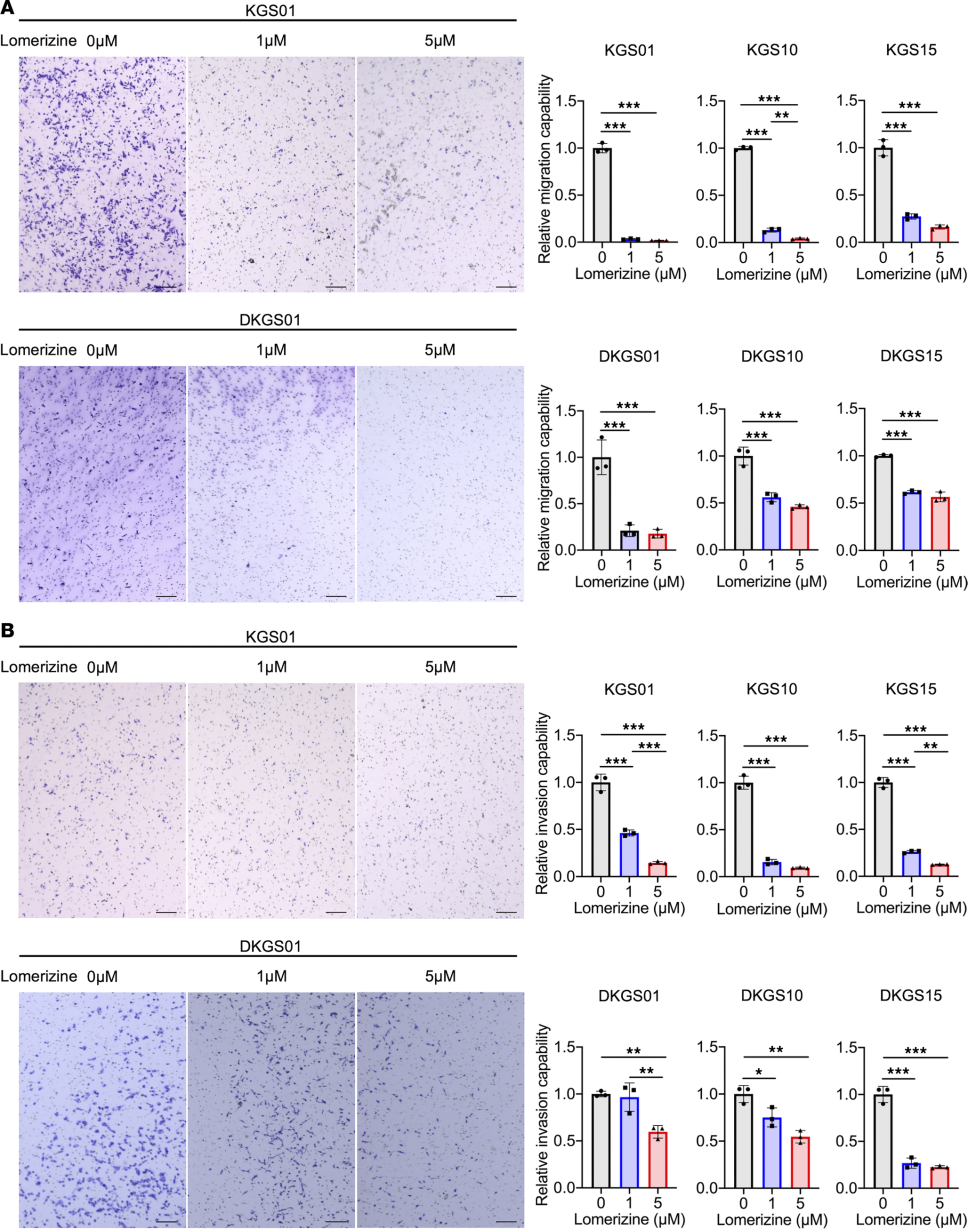
Effects of lomerizine on migration and invasion of glioblastoma cells. The experiments were performed in Transwell chambers with non-Matrigel-coated or Matrigel-coated membranes containing 3 GICs (KGS01, KGS10, and KGS15) and their differentiated cells (DKGS01, DKGS10, and DKGS15). The number of migrated/invaded cells was counted in 9 randomly chosen high-power fields (HPFs) per treatment group, and the means from 3 wells were calculated. (**A**) Representative images of the Transwell migration assay of KGS01 and differentiated KGS01. Glioma cells, including GICs, were treated with lomerizine at 0 (DMSO), 1, and 5 μM. Cells migrating through a non-Matrigel-coated Transwell chamber were scored in the presence and absence of lomerizine for 12 hours. Bar graphs revealing the average number of migrated glioma cells per HPF for all GICs (KGS01, KGS10, and KGS15) and their differentiated cell lines (DKGS01, DKGS10, and DKGS15). (**B**) Representative images of the Transwell invasion assay of KGS01 and differentiated KGS01. Glioma cells were treated with lomerizine at 0 (DMSO), 1, and 5 μM. Cells invading the Matrigel-coated Transwell chamber were scored in the presence and absence of lomerizine for 12 hours. Bar graphs revealing the average number of invaded glioma cells per HPF for all GICs and their differentiated cell lines. Scale bars: 200 μm (**A** and **B**). Data were analyzed by 1-way ANOVA with Tukey’s multiple-comparison test. *P < 0.05, **P < 0.01, ***P < 0.005 vs. DMSO.

To evaluate the effect of lomerizine on glioma cell migration, we performed Boyden chamber migration assays for all cell lines. Migration and invasion were evaluated within 12 hours of exposure to lomerizine with no significant difference in cell viability (Supplemental Figure 4). We found that lomerizine inhibited Transwell migration of KGS01 cells by 97% at 1 μM and 98% at 5 μM, of KGS10 cells by 87% at 1 μM and 96% at 5 μM, and of KGS15 cells by 74% at 1 μM and 87% at 5 μM (Figure 4A). Furthermore, to investigate the invasion activity, we evaluated Matrigel invasion. Lomerizine inhibited the Matrigel invasion of KGS01 cells by 54% at 1 μM and 85% at 5 μM, of KGS10 cells by 84% at 1 μM and 91% at 5 μM, and of KGS15 cells by 74% at 1 μM and 87% at 5 μM (Figure 4B). Lomerizine also reduced the migration and invasion capacities of all differentiated GICs and all glioma cell lines; these effects were particularly pronounced in GICs (Figure 4, A and B, and Supplemental Figures 5 and 6). These results suggested that lomerizine inhibited cell migration and invasion in both GICs and differentiated glioma cells.

### Lomerizine decreases GIC stemness by downregulating SOX2 expression.

**Figure 5:**
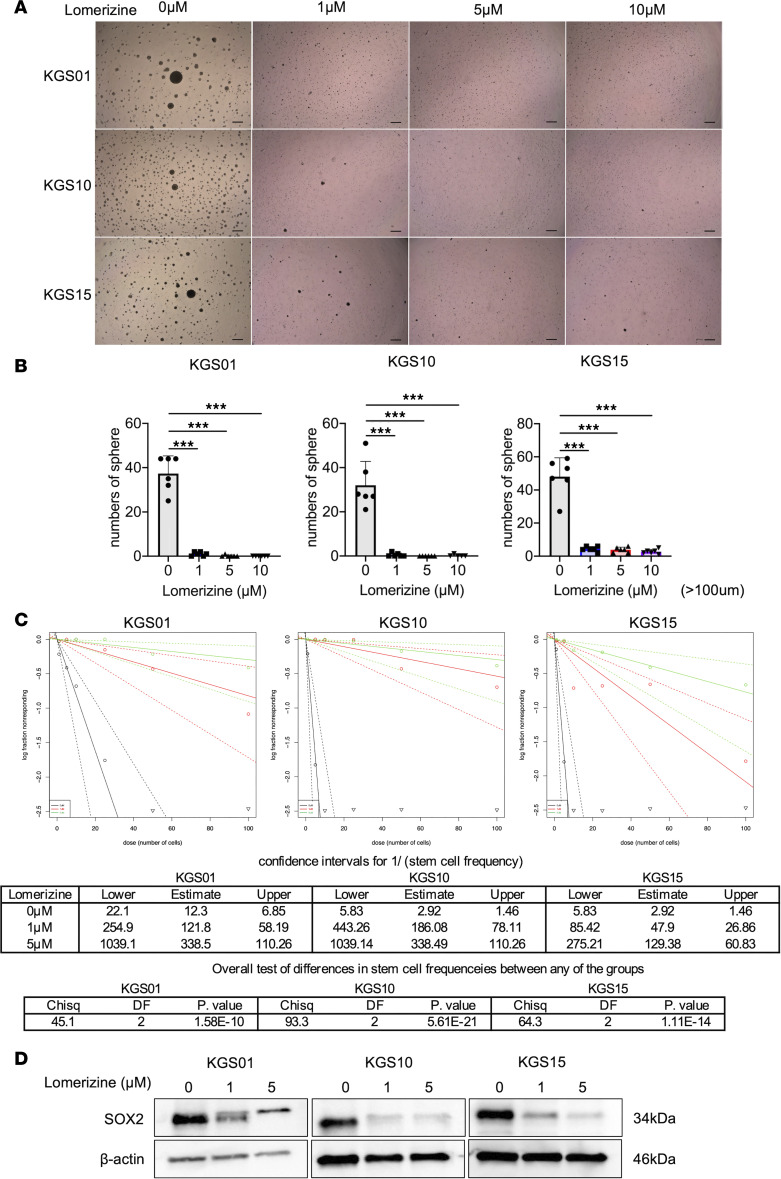
Effects of lomerizine on stemness in GICs. (**A** and **B**) The sphere-forming assay was performed using GICs (KGS01, KGS10, and KGS15). Sphere numbers were counted 7 days after treatment with lomerizine at 0 (DMSO), 1, and 5 μM. Each experiment was repeated at least 3 times. (**A**) Representative images of the sphere-forming assay 7 days after treatment with lomerizine in GICs. (**B**) Bar graphs indicating the number of spheres with diameters larger than 100 μm in each GIC. (**C**) In vitro analysis of GIC self-renewal using the limiting dilution assay (LDA). LDA data analysis by the Extreme Limiting Dilution Analysis (ELDA) tool. The number of initially seeded cells (*x* axis) is plotted against the log fraction of nonresponders corresponding to wells without any detected sphere (*y* axis). The slope of the line represents the log active cell fraction. The dashed lines give the 95% confidence interval. (**D**) Western blot results showing the expression of sex-determining region Y box 2 (SOX2) in GICs treated with lomerizine for 48 hours. β-Actin was used as a loading control in Western blotting analysis. Scale bars: 100 μm (**A**). Bars represent mean values ± SD (**B**). Data were analyzed by 1-way ANOVA with Tukey’s multiple-comparison test. ***P < 0.005 vs. DMSO.

We evaluated the effect of lomerizine on the maintenance of GIC stemness by conducting sphere formation assays. Lomerizine treatment reduced the number of GIC spheres, and a few spheres were formed at very low drug doses (Figure 5, A and B). We also performed a sphere limiting dilution assay followed by quantification with the Extreme Limiting Dilution Analysis (ELDA) tool to investigate the lomerizine effect on self-renewal ability of GICs. As a result, lomerizine led to drastic inhibition in sphere number formation in all GICs (Figure 5C). Furthermore, SOX2 expression was suppressed by lomerizine in a dose-dependent manner in all GIC lines (Figure 5D). These results suggest that lomerizine decreases the tumorigenic features of GICs by downregulating SOX2 protein expression.

### Lomerizine suppresses STAT3 activation in glioma cells.

**Figure 6:**
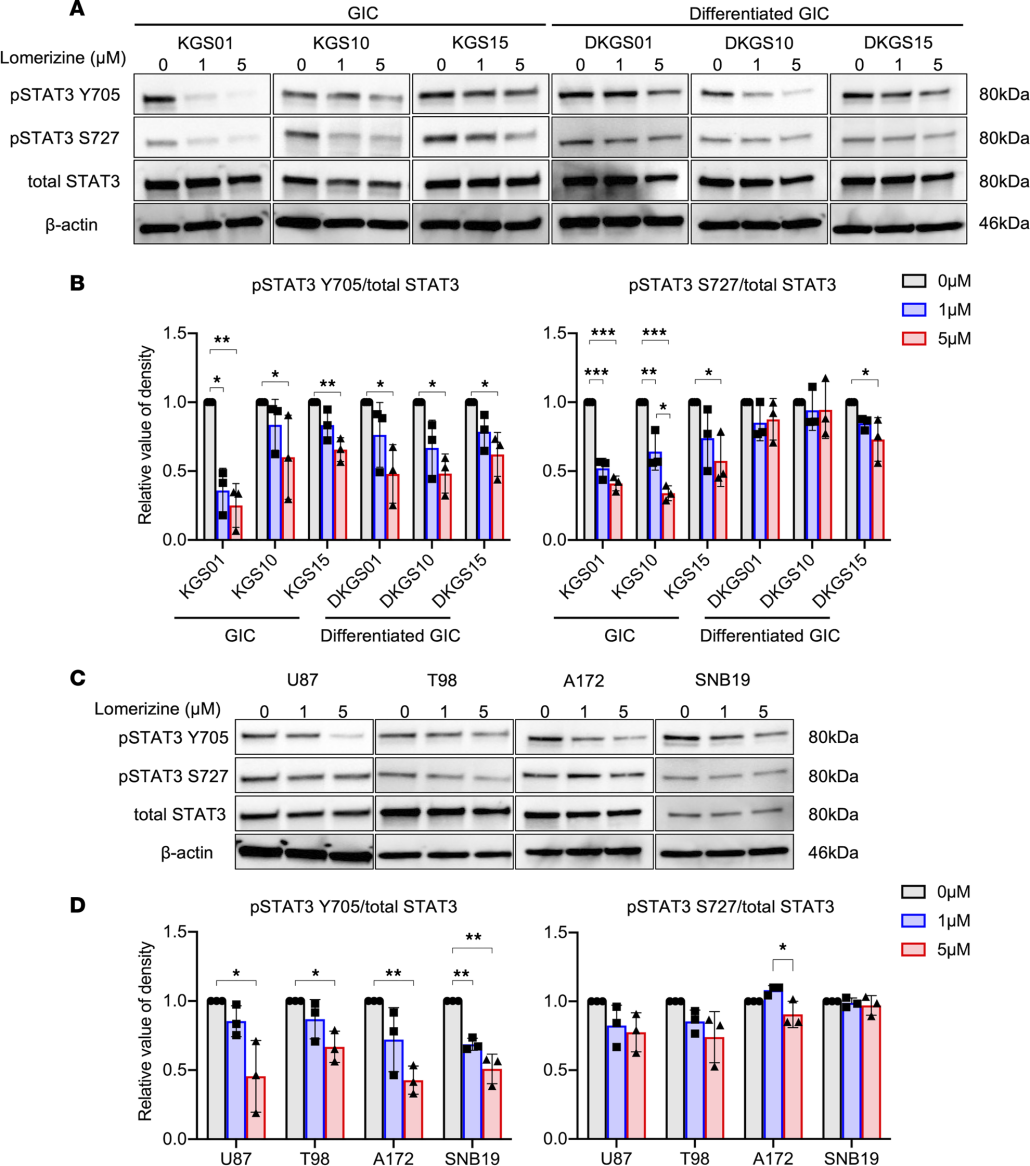
Effects of lomerizine on the STAT3 signaling pathway of glioblastoma cells. Western blotting of 3 GICs (KGS01, KGS10, and KGS15) and their differentiated cells (DKGS01, DKGS10, and DKGS15) (**A** and **B**) and 4 human glioma cell lines (U87, T98, A172, and SNB19) (**C** and **D**) treated with lomerizine. All glioma cells were treated with lomerizine at 0 (DMSO), 1, and 5 μM. Western blotting was performed on each cell line after 12 hours of exposure. The analyzed proteins included STAT3, phospho-STAT3 Y705, and phospho-STAT3 S727. The relative levels of protein expression were normalized to β-actin, serving as an internal control. The band intensity was analyzed via Western blotting using a CS analyzer. (**A**) Western blotting results for expression of STAT3, p-STAT3 Y705, and p-STAT3 S727 in the 3 GICs and their differentiated cells. Expression of p-STAT3 Y705 was suppressed in all GICs and their differentiated cells. Furthermore, expression of p-STAT3 S727 was suppressed in all GICs and in partially differentiated GICs. (**B**) The average phosphorylation ratio of STAT3 (tyrosine 705 and serine 727) expression in the 3 GIC cell lines and their differentiated cells was quantified using Western blotting. (**C**) Western blotting results for the expression of STAT3, p-STAT3 Y705, and p-STAT3 S727 in the 4 human glioma cell lines. Expression of p-STAT3 Y705 was suppressed in all the glioma cell lines. In contrast, expression of p-STAT3 S727 was suppressed only in T98 cells. (**D**) The average phosphorylation ratios of STAT3 (tyrosine 705 and serine 727) in the 4 glioma cell lines were quantified using Western blotting. Error bars represent the SD for each protein in individual cell groups from 3 separate experiments (**B** and **D**). Data were analyzed by 1-way ANOVA with Tukey’s multiple-comparison test (3 independent experiments). *P < 0.05, **P < 0.01, ***P < 0.005 vs. DMSO.

**Figure 7:**
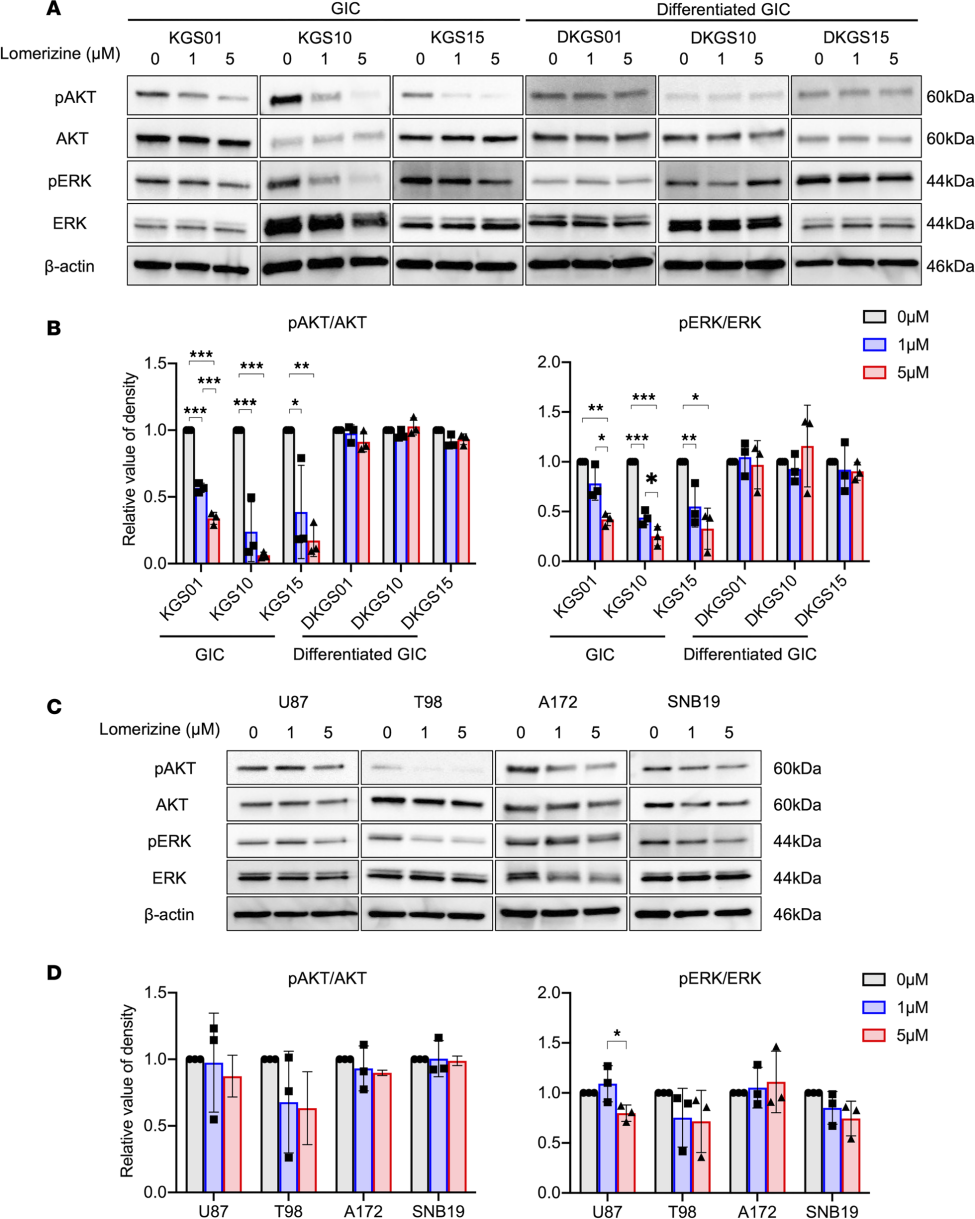
Effects of lomerizine on the AKT and ERK signaling pathway of glioblastoma cells. Western blotting of 3 GICs (KGS01, KGS10, and KGS15) and their differentiated cells (DKGS01, DKGS10, and DKGS15) (**A** and **B**) and 4 human glioma cell lines (U87, T98, A172, and SNB19) (**C** and **D**) treated with lomerizine. All glioma cells were treated with lomerizine at 0 (DMSO), 1, and 5 μM. Western blotting was performed on each cell line after 12 hours of exposure. The analyzed proteins included AKT, ERK, phospho-AKT (p-AKT), and phospho-ERK (p-ERK). The relative levels of protein expression were normalized to β-actin, serving as an internal control. The band intensity was analyzed via Western blotting using a CS analyzer. (**A**) Western blotting results for expression of AKT, p-AKT, ERK, and p-ERK in the 3 GICs and their differentiated cells. Expression of p-AKT and p-ERK was suppressed in all GICs and in partially differentiated GICs. (**B**) The average phosphorylation ratio of AKT and ERK expression in the 3 GIC cell lines and their differentiated cells was quantified using Western blotting. (**C**) Western blotting results for expression of AKT, p-AKT, ERK, and p-ERK in the 4 human glioma cell lines. Expression of p-AKT and p-ERK was partially suppressed in glioma cell lines. (**D**) The average phosphorylation ratios of AKT and ERK in the 4 glioma cell lines were quantified using Western blotting. Error bars represent the SD for each protein in individual cell groups from 3 separate experiments (**B** and **D**). Data were analyzed by 1-way ANOVA with Tukey’s multiple-comparison test (3 independent experiments). *P < 0.05, **P < 0.01, ***P < 0.005 vs. DMSO.

Western blotting was performed to examine the effects of lomerizine on the activation of STAT3, AKT, and ERK, as the pathways involving these proteins have been implicated in glioma cell proliferation. Lomerizine dose-dependently suppressed the expression of phospho-STAT3 Y705 in GICs, differentiated GICs, and 4 human glioma cell lines (U87, T98, A172, and SNB19) (Figure 6, A–D). Lomerizine also dose-dependently suppressed the expression levels of phospho-STAT3 S727, phospho-AKT, and phospho-ERK in GICs but not in their differentiated cell lines and 4 human glioma cell lines (Figure 7, A–D). Thus, lomerizine alters STAT3 signaling and inhibits cell proliferation, migration, and invasion. In GICs, it exerts a strong antitumor effect by altering AKT and ERK signaling. The inhibitory effects of lomerizine on proliferation, migration, and invasion-related signal transduction were maintained for more than 24 hours in almost all GICs and human glioma cell lines (Supplemental Figure 7).

In differentiated glioma cells, which have little effect on AKT and ERK signaling by lomerizine, to confirm the importance of STAT3 inhibition for lomerizine, we performed Alamar blue proliferation assay using lomerizine, two different STAT3 siRNAs, and their combination (Supplemental Figure 8A). Lomerizine showed only a little additive effect on the cell growth inhibition in combination with STAT3 siRNA treatment (Supplemental Figure 8B). Therefore, the major mechanism of the antitumor effect of lomerizine in differentiated glioma cells appears to be the inhibition of STAT3 activation.

### Lomerizine reduces tumor size and prolongs survival of a xenograft mouse model.

**Figure 8:**
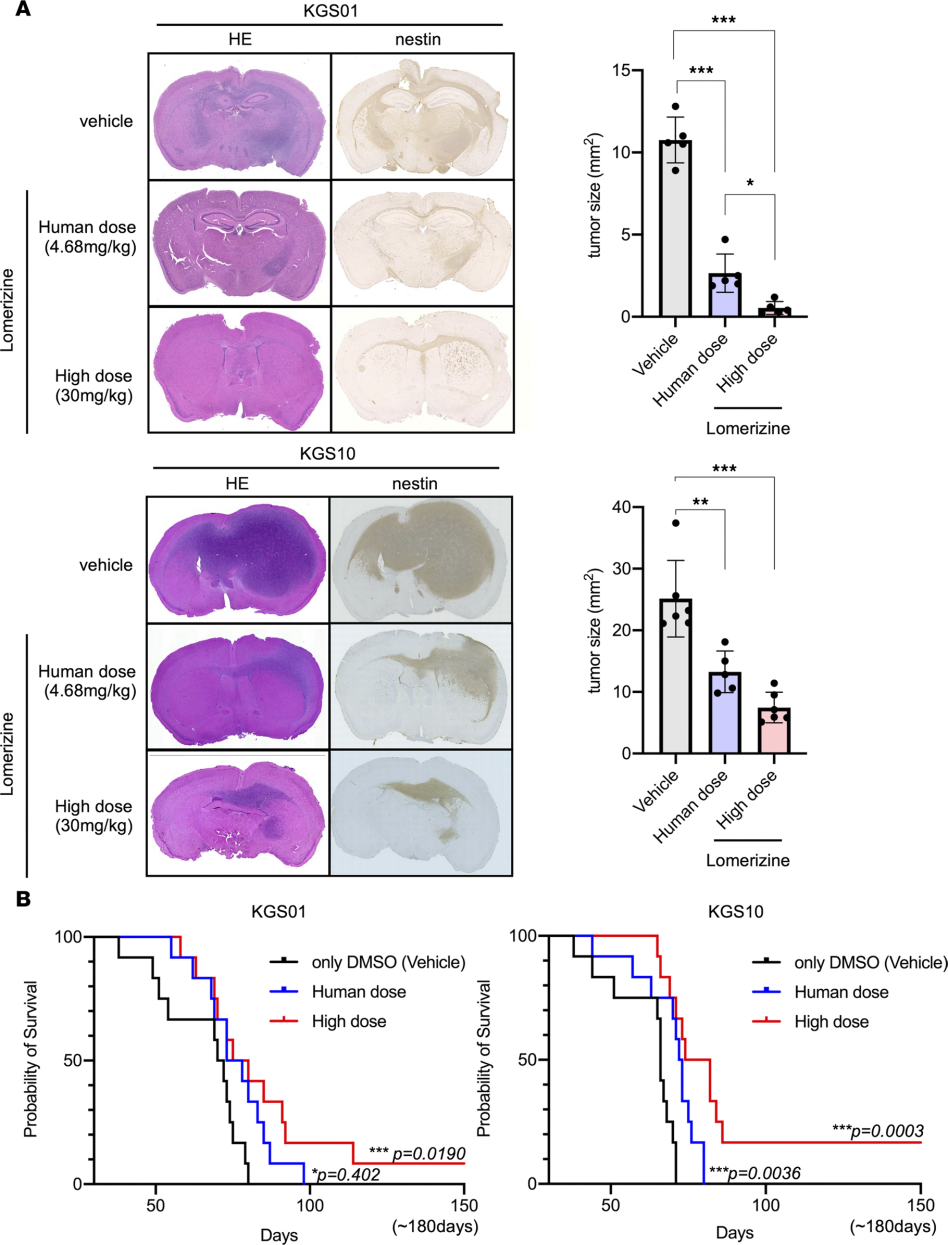
Effects of lomerizine treatment on the xenograft mouse model in vivo. We generated a mouse brain tumor model by transplanting KGS01 and KGS10 cells into the brain, and the mice were randomly assigned to receive human-dose lomerizine (4.68 mg/kg), high-dose lomerizine (30 mg/kg), or vehicle (DMSO) through daily oral administration. (**A**) Hematoxylin and eosin staining (left column) and immunohistochemistry for nestin (right column in serial coronal sections of xenograft mouse brains 55 days after lomerizine treatment. Bar graphs indicating the tumor size (mm^2^) of mice in each treatment group. (**B**) Overall survival of mice treated with lomerizine and DMSO (control). Lomerizine significantly prolonged the survival of KGS01 and KGS10 mice in a dose-dependent manner. Log-rank test: KGS01, *P* = 0.402 (human-dose lomerizine group) and 0.0190 (high-dose lomerizine group); KGS10, *P* = 0.036 (human-dose lomerizine group) and 0.0003 (high-dose lomerizine group). Scale bars: 100 μm (**A**). Bars represent mean values ± SD (**A**). Data were analyzed by 1-way ANOVA with Tukey’s multiple-comparison test (**A**) and log-rank test (**B**). *P < 0.05, **P < 0.01, ***P < 0.005 vs. vehicle.

GBM is an extremely heterogeneous disease, so we used two GIC cell lines, KGS01 and KGS10, and established mouse models to investigate lomerizine’s efficacy with different molecular subtypes in vivo. We have previously reported that KGS01 highly expresses the mesenchymal marker CD44, while KGS10 highly expresses the proneural marker OLIG2, indicating that these cell lines belong to different molecular subtypes (25). To verify our previous findings in vitro, KGS01 and KGS10 (1 × 10^6^ cells) were injected into the mouse brain and treated with 2 different concentrations of lomerizine (human dose, 4.68 mg/kg; high dose, 30 mg/kg) or vehicle (only DMSO). No apparent adverse events were caused by lomerizine treatment in this experiment (Supplemental Figure 9A). In both mouse models, the tumor size in the vehicle group tended to be significantly larger than that in the lomerizine-treated group and was reduced in a concentration-dependent manner (Figure 8A). To assess the long-term effects of lomerizine, KGS01 and KGS10 were implanted into a cohort of 36 mice for survival analysis. Consequently, the median survival times of mice treated with lomerizine were 75.5 days (KGS01, *P* = 0.0402) and 72.5 days (KGS10, *P* = 0.0036) in the human-dose group and at least 77.5 days (KGS01, *P* = 0.0190) and 78.0 days (KGS10, *P* = 0.003) in the high-dose group, compared with 71.0 days (KGS01) and 66.0 days (KGS10) in the vehicle group (Figure 8B). In the high dose lomerizine treatment group, 1 mouse (8.3%) of KGS01 mouse model and 2 mice (17%) of KGS10 mouse model survived beyond 150 days, which was more than twice the survival rate of the vehicle group, and were sacrificed to confirm tumor formation. Both the human-dose and high-dose lomerizine treatment groups showed statistically significant prolongation of median survival.

**Figure 9:**
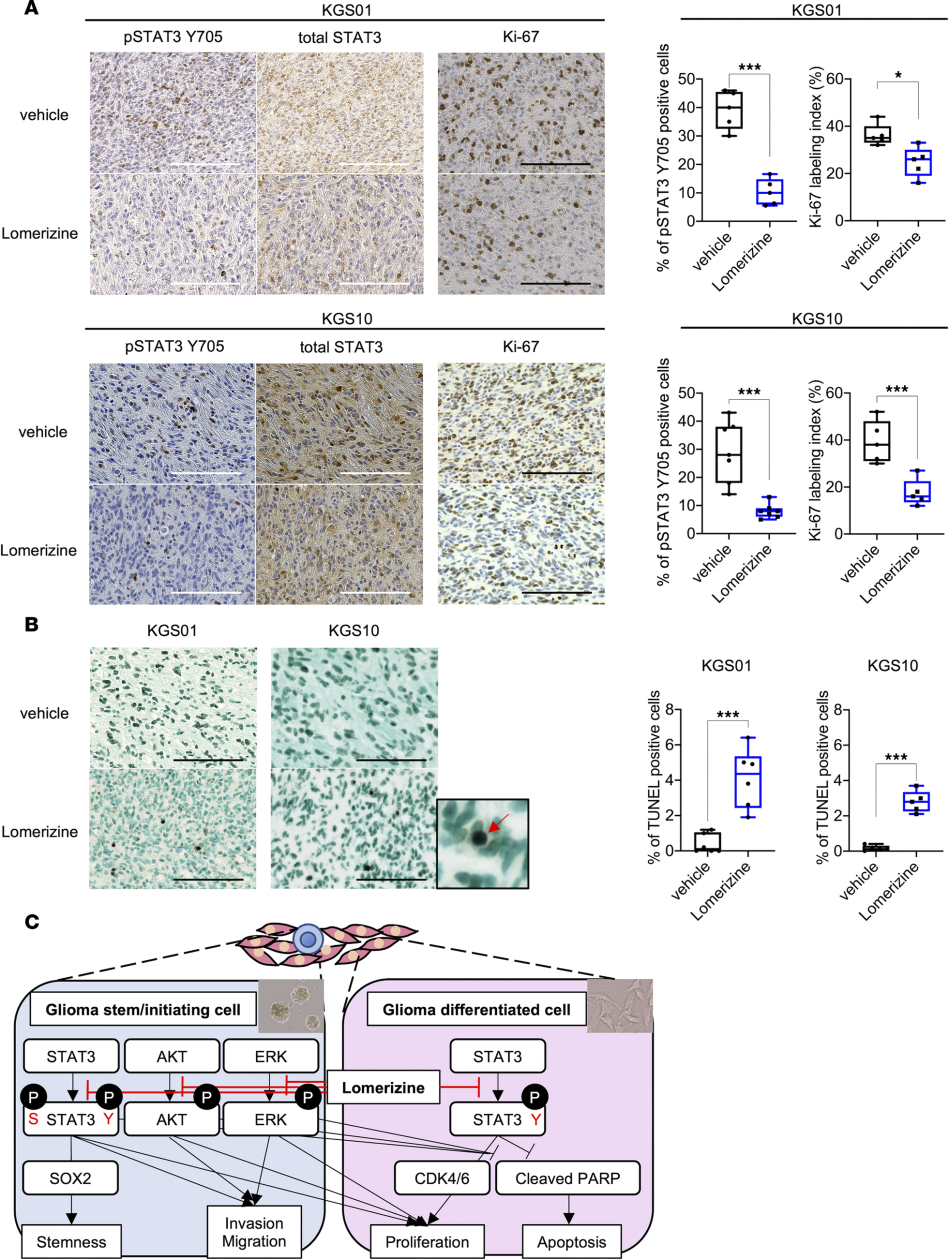
Effects of lomerizine treatment on cell signaling pathways in vivo. (**A**) Top panels: Representative immunohistochemical sections of the KGS01 mouse models showing phosphorylated STAT3 at tyrosine 705 and Ki-67 in untreated and human-dose lomerizine–treated brain tumors. Bar graph showing the percentage of p-STAT3 Y705–positive cells in each treatment group (left) and the immunoactivity of Ki-67 in each treatment group (right). Bottom panels: Representative immunohistochemical sections of the KGS10 mouse model showing phosphorylated STAT3 at tyrosine 705 and Ki-67 in untreated and human-dose lomerizine–treated brain tumors. Bar graph showing the percentage of p-STAT3 Y705–positive cells in each treatment group (left) and the immunoactivity of Ki-67 in each treatment group (right). (**B**) Representative immunohistochemical sections after TUNEL staining showing apoptotic cells. Inset indicating an enlarged image of cells undergoing apoptosis (stained black). Red arrows indicate the cells in the apoptotic state. Inset shows higher magnification image (4×) of apoptotic cell. Bar graph showing the number of apoptotic cells in each treatment group. (**C**) Schematic representation of the function of lomerizine in GICs and glioma cells. Bars represent mean values ± SD (**A** and **B**). Scale bars: 100 μm (**A** and **B**). Data were analyzed by 2-tailed Student’s *t* test. *P < 0.05, ***P < 0.005 vs. vehicle.

To examine the antitumor effect of lomerizine on GBM in detail, immunostaining was performed in the vehicle group and the human-dose lomerizine treatment group. To evaluate the changes in molecular signaling after lomerizine treatment, the expression levels of phospho-STAT3 Y705 in each treatment group were investigated. The results showed that phospho-STAT3 Y705 was spot-positive, and the number of phospho-STAT3 Y705–positive cells in the lomerizine-treated group was significantly lower than that in the vehicle group (Figure 9A). Next, to examine the proliferative potential of brain tumors, slide sections from each group were stained with Ki-67, revealing that the Ki-67 labeling index in the lomerizine-treated group was significantly lower than that in the vehicle group (Figure 9A). Finally, apoptotic cells in the tumor tissues were evaluated by TUNEL staining. The number of black-stained apoptotic cells was significantly higher in the lomerizine-treated group than in the vehicle group (Figure 9B). Thus, lomerizine induced apoptosis by decreasing phosphorylated STAT3 Y705 in vivo, resulting in a strong anti-GBM effect.

## Discussion

This is, to our knowledge, the first report demonstrating the dual antitumor effect of the anti-migraine drug lomerizine on both GICs and differentiated glioma cells. Furthermore, these effects were observed in all 10 examined glioma cell lines, including GICs and their differentiated cells. Considering that the BBB poses a serious challenge in drug repurposing for intracranial tumors, the fact that lomerizine is already known to cross the BBB constitutes a major advantage. In summary, lomerizine inhibited proliferation, migration, and invasion by altering STAT3 signaling, inducing apoptosis, and inducing G_1_ cell cycle arrest in all glioma stem and differentiated cell lines (Figure 9C). Lomerizine also decreased stemness and had a more aggressive antitumor effect by downregulating AKT and ERK signaling in GICs. Collectively, these findings suggested that lomerizine potentially reduced GBM growth and prevented relapse.

In this study, several T-type calcium channel blockers showed antitumor effects against GICs. Lomerizine is a particularly potent inhibitor of T-type calcium channels as a prophylactic agent for migraine (IC_50_, 0.46 nM) (26). In a previous report, the T-type calcium channel Ca_v_3.2 was markedly upregulated in GICs compared with differentiated glioma cells; was strongly implicated in the proliferation, survival, and stemness of GICs; and suppressed GIC growth by inhibiting the AKT pathway (27). We also investigated the differences in the expression of genes encoding T-type calcium channels in GICs and their differentiated cells using quantitative reverse transcription PCR. T-type calcium channels Ca_v_3.1, Ca_v_3.2, and Ca_v_3.3 were highly expressed in GICs, consistent with the strong antitumor effect of lomerizine in GICs (Supplemental Figure 10A). Subsequently, T-type calcium channel activity was directly assessed in GICs using whole-cell patch-clamp recordings (Supplemental Figure 10, B and C). Voltage-dependent inward currents were evoked by a series of depolarizing steps from a holding potential of –90 mV, with 10 mM Ba^2+^ used as the charge carrier. The currents were activated at lower membrane potentials and exhibited rapid inactivation; less current was elicited by a depolarizing pulse from a shallow holding potential (–60 mV). These characteristics are consistent with the typical properties of T-type calcium channels. Furthermore, we performed calcium imaging experiments using Fluo-4 AM to investigate the effect of lomerizine on T-type calcium channel activity in GICs. SAK3 (1 nM), a T-type calcium channel activator (28), increased intracellular calcium levels across all 3 cell lines; however, this increase was suppressed in the presence of lomerizine (Supplemental Figure 10, D and E). These results indicate that lomerizine exerts inhibitory effects on T-type calcium channels in GICs. At last, we investigated whether the antitumor effect of lomerizine against GBM cells is a result of the inhibition of T-type calcium channels by using 2 drugs, SAK3 and ZSET1446, which are typical T-type calcium channel enhancers (29). As a result, typical T-channel activators partially rescued lomerizine-induced inhibition of cell proliferation and restored the reduction in phosphorylation of STAT3 at Y705 caused by lomerizine (Supplemental Figure 11). These results suggest that lomerizine exhibits potent antitumor effects against glioblastoma cells, particularly GICs, by inhibiting T-type calcium channels.

STAT3 is a stemness-associated transcription factor that is activated by the phosphorylation of tyrosine 705, following which it is rapidly translocated into the nucleus to regulate target genes. In high-grade glioma, phospho-STAT3 Y705 is expressed in more than 80% of cases (30) and is implicated in glioma cell hyperproliferation and anti-apoptosis (31, 32). In addition, phospho-STAT3 Y705 promotes glioma cell migration and invasion (33, 34). In fact, the expression of phospho-STAT3 Y705 is clinically correlated with GBM prognosis (35). Therefore, phospho-STAT3 Y705 is a promising molecular target for the treatment of GBM. On the other hand, although several STAT3 inhibitors demonstrated potent anti-GBM effects including on GICs in vitro, their efficacy was inadequate in intracranial mouse tumor models, and poor drug permeability into tumors within the central nervous system was considered a major obstacle to treatment (36). In this study, lomerizine downregulated the expression of phospho-STAT3 Y705 in GICs and all other differentiated glioma cell lines in culture as well as in a xenograft mouse model. As a result, oral administration of lomerizine reduced the tumor size and prolonged the survival of a mouse model established by injection of KGS01 and KGS10 cells. Several xenograft organ pathological evaluations were performed to investigate adverse effects of lomerizine treatment, but no apparent adverse events were shown. We also undertook proliferation assay using HFF-1, a normal human fibroblast cell line, and no significant difference was observed following lomerizine treatment (Supplemental Figure 9B). STAT3 is also essential for the maintenance of stemness by promoting the expression of the GIC stemness factors SOX2, nestin, and CD133 in glioma stem cells (37, 38). Glioma stem cells are evidently resistant to therapy (39), and the expression of SOX2, a key stem cell marker, enhances radiation and chemotherapy resistance (40). In this study, lomerizine also suppressed the STAT3/SOX2 pathway, which is critical for maintaining stemness in glioma stem cells and inhibited tumorigenesis in vitro and in vivo. Lomerizine, which has a potent antitumor effect against GICs, has the potential to improve chemo- and/or radioresistance in gliomas, although further investigations are warranted.

Lomerizine treatment of GICs also downregulates the expression of activated AKT and ERK, which are kinases involved in proliferation, invasion, and migration (41–44). Currently, 4 subtypes of GBM can exist in the same individual — classical, mesenchymal, neural, and proneural — making GBM treatment more complex and challenging (45). Each of these subtypes expresses different signaling markers that alter downstream signaling pathways. For example, phospho-STAT3 Y705 is highly expressed in mesenchymal GBM, whereas its expression is low in proneural GBM; conversely, AKT is highly expressed in proneural-type GBM (46). Therefore, it would be ideal to simultaneously suppress multiple signaling pathways. Lomerizine has a suppressive effect on STAT3, AKT, and ERK in GICs, can flexibly respond to cell-specific signal transduction, and has an antitumor effect. One possible mechanism by which lomerizine inhibited multiple signaling pathways is through the inhibition of T-type calcium channels. In malignant tumors, various calcium signals have been reported to activate cell signaling pathways involved in tumor proliferation, including AKT, ERK, and STAT3 (47, 48). T-type calcium channels are no exception. Inhibition of T-type calcium channels had been shown to suppress the AKT, ERK, and STAT3 pathways in multiple cancers, including glioblastoma (27, 49, 50). The inhibition of dual or multiple signaling pathways has strong antitumor effects against GBM and a variety of other carcinomas. For example, the inverse correlation of expression between MEK/ERK signaling and STAT3 signaling leads to drug resistance in pancreatic ductal adenocarcinoma; this resistance was overcome and a strong antitumor effect was demonstrated by the combined inhibition of MEK and STAT3 (51). In addition, STAT3 is involved in the resistance to PI3K/AKT/mTOR inhibitors in PTEN-deficient cancer cells, and combination therapy with PI3K/AKT/mTOR and STAT3 inhibitors has strong antitumor effects both in vitro and in vivo (52). Thus, intracellular signaling is extremely delicate and intricately interrelated, and the inhibition of multiple signaling pathways may achieve antitumor effects beyond an additive relationship. Polypharmacological multitargeted therapy using a single agent is superior to drug combination therapy in terms of drug dosage adjustment and minimizing of side effects arising from drug-drug interactions. It is also more likely to attain the desired selective profile (53). In this regard, lomerizine is an ideal anti-glioma agent because of its inhibitory effect on 3 major pathways involved in cell proliferation against GICs as a single agent. Furthermore, the inhibitory effect of lomerizine on signaling associated with proliferation, migration, and invasion was maintained for more than 24 hours in almost all GIC and human glioma cell lines. Lomerizine is a drug usually administered twice daily, and its sustained effects on signaling and tumor suppression can be expected when it is continued with the usual oral dosage for migraine. In fact, daily oral administration of lomerizine has potent antitumor effects against multiple subtypes of GBM as a single agent. These results suggest that lomerizine is an ideal treatment option for GBM and shows promising potential for overcoming treatment resistance. Lomerizine produced favorable outcomes in multiple subtypes of GBM cells in vivo, including prolonged overall survival, suggesting that it enhances the potential to suppress the progression of GBM in patients in clinical practice.

In conclusion, this study demonstrates that lomerizine can suppress GBM growth both in vitro and in vivo, suggesting its potential for clinical application in improving patient prognosis.

## Methods

### Sex as a biological variable.

KGS01 and KGS15 was established by male patients and KGS10 was established by female patients. In the animal experiments, our study exclusively examined female mice; it is unknown whether the findings are relevant to male mice. Since our laboratory has previously established a brain tumor model using female mice, we used only female mice in this experiment.

### Cell culture.

Human patient-derived GIC lines KGS01, KGS10, and KGS15 were established at Kanazawa University according to the protocols approved by the ethics committee of Kanazawa University. KGS01, KGS10, and KGS15 were confirmed as GIC lines, consistent with previous reports (13, 25). Briefly, these cells have the ability to form spheres and express surface markers characteristic of stemness such as CD133, CD44, and nestin (Supplemental Figure 12A). They also differentiate into astrocyte-like cells positive for glial fibrillary acidic protein (GFAP) and oligodendrocyte transcription factor 2 (Olig2), as well as into neuron-specific class III β-tubulin–positive (Tuj1-positive) neuron-like cells, in Dulbecco’s modified Eagle medium (DMEM)/F12 (Thermo Fisher Scientific) supplemented with 10% FBS (Supplemental Figure 11A) (13, 25). All GIC lines were cultured in neurosphere formation medium containing DMEM supplemented with 20 ng/mL recombinant human epidermal growth factor (EGF; Sigma-Aldrich), 20 ng/mL recombinant human basic fibroblast growth factor (bFGF; Sigma-Aldrich), MACS NeuroBrew-21 supplement without vitamin A (Miltenyi Biotec), GlutaMAX (Thermo Fisher Scientific), and 1% penicillin/streptomycin (Thermo Fisher Scientific).

Human glioma cell lines U87, T98, A172, and SNB19 were purchased from the American Type Culture Collection (ATCC, Manassas, Virginia) using short tandem repeat profile analysis. In this study, we established differentiated KGS01 (DKGS01), differentiated KGS10 (DKGS10), and differentiated KGS15 (DKGS15) cell lines, which were then cultured under the same conditions as the human GBM cell lines. We confirmed the differentiation of GICs by suppressing the expression of SOX2, a transcription factor involved in the maintenance of stem cell characteristics (Supplemental Figure 12B). Authentication of the cell lines was unnecessary because they were expanded after fewer than 2 passages and stored at –80°C. Low-passage cells were used for all experiments within 6 months of resuscitation. These cells were cultured at 37°C under a humidified 5% CO_2_ atmosphere in DMEM supplemented with 10% heat-inactivated FBS (Cosmo Bio Co. Ltd.) and 1% penicillin/streptomycin (Thermo Fisher Scientific).

### Drug screening.

Candidate anti-GBM drugs were identified by screening drug libraries, which contain 1,301 diverse chemical compounds (FDA Approved Drug Library [640 compounds; ENZO; CB-BML-2841J0100], ICCB Known Bioactive Library [480 compounds; ENZO; CB-BML-2840J0100], Kinase Inhibitor Library [80 compounds; ENZO; CB-BML-2832J0100], Fatty acid Drug Library [68 compounds; ENZO; CB-BML-2803J0100], Phosphatase Inhibitor Library [33 compounds; ENZO; CB-BML-2834J0100]. A 3-step screening was performed to identify effective compounds against GICs. First, we treated 3 GICs with each compound at 3 concentrations (1, 5, and 20 μM) in a 384-well plate (Corning) for 48 hours, followed by measurement of viable cell number using the 2-(2-methoxy-4-nitrophenyl)-3-(4-nitrophenyl)-5-(2,4-disulfophenyl)-2*H*-tetrazolium (WST-8) assay (DOJINDO Laboratories), as previously described (13). Compounds producing a dose-dependent reduction in cell number peaking at ≥25% were selected. Second, drugs previously reported to have therapeutic potential against GBM and those in clinical trials were excluded. Third, candidate compounds that were already in regulatory approval in Japan were detected. At last, Alamar blue assays (Bio-Rad Laboratories) were performed using the 2 GIC lines (KGS01 and KGS10) with lower concentrations (0.5, 1, and 5 μM) of the selected compounds (details provided below). Candidate drugs that potently inhibited cell viability even at the lowest concentrations were selected.

### Cell proliferation and viability assay, and measurement of IC_50_.

Cell proliferation and viability of all glioma cell lines were assessed using the Alamar blue assay (Bio-Rad Laboratories). We first dissociated patient-derived GIC spheres into single cells using StemPro Accutase (Thermo Fisher Scientific) and reseeded these cells onto 96-well Costar ultra-low-attachment plates (Corning) at a density of 3.0 × 10^3^ cells per 200 μL. Human glioma cell lines and differentiated GICs were dissociated into single cells using 0.05% trypsin-EDTA (Thermo Fisher Scientific) and then seeded onto 96-well flat-bottomed tissue culture plates (Corning) at a density of 1.0 × 10^3^ cells per 200 μL of culture medium supplemented with 0.5% FBS. The cells were treated with different concentrations of lomerizine (1, 5, or 10 μM) or dimethylsulfoxide (DMSO), followed by incubation for 3 hours with Alamar blue (20 μL). Furthermore, we performed a cell proliferation assay with pretreatment using two T-type calcium channel enhancers, SAK3 (Sigma-Aldrich) and ZSET1446 (TargetMol), to investigate the involvement of T-type calcium channel activity in the cell proliferation inhibitory effect of lomerizine (28, 29). SAK3 and ZSET1446 were prepared at a final concentration of 1 nM and added 2 hours before lomerizine (5 μM) treatment. The relative number of viable cells was determined by measurement of the absorbance using a microplate reader (Bio-Rad Laboratories) at 0, 24, 48, and 72 hours after lomerizine addition (measurement, 590 nm; reference, 540 nm). The average absorbance values from 3 wells in each treatment group were calculated and plotted successively for the proliferation assay. The values of half-maximal inhibitory concentration (IC_50_) were calculated using log (inhibitor) versus response including variable slope (4 parameters) statistics and normalized in GraphPad Prism 8 (Dotmatics).

### Apoptosis assays.

Immunofluorescent staining was performed to detect apoptosis in the presence of lomerizine. Briefly, differentiated GICs and human glioma cell line were grown on glass coverslips in 6-well flat-bottomed tissue culture plates (Corning) at a density of 1.0 × 10^5^ cells per 1,000 μL. GICs were also grown on laminin-coated glass coverslips in 6-well flat-bottomed tissue culture plates (Corning) at a density of 3.0 × 10^5^ cells per 1,000 μL. All cells were treated with lomerizine (1 or 5 μM) or DMSO as a control for 24 hours, washed 3 times in PBS, and stained with propidium iodide (PI) and Hoechst 33258 (DOJINDO Laboratories). The cell status was defined by the staining pattern. Viable cell nuclei were stained blue with Hoechst 33258, and the cytoplasm was stained red with PI, while apoptotic cell nuclei were pink owing to costaining with Hoechst 33258 and PI (16, 54). The average number of cells undergoing apoptosis in 6 high-power fields (HPFs) per treatment group was calculated and plotted.

The annexin V assay was performed using an Annexin V–FITC detection kit, according to the manufacturer’s protocol (Abcam). Briefly, GIC cells, differentiated GICs, and human glioma cell lines were seeded at a density of 1 × 10^5^ cells per 2 mL per well on 6-well plates with culture medium containing lomerizine (1 or 5 μM) or DMSO. After 24 hours of treatment, annexin V–FITC–positive cells were counted through fluorescence-activated cell sorting using a FACSCanto II system (Becton, Dickinson and Co.) and FlowJo software (Becton, Dickinson and Co.). Cells positive for annexin V–FITC but negative for PI were considered as early apoptotic cells, and double-positive cells were considered late apoptotic cells. The average of the percentage of early apoptosis, the percentage of late apoptosis, and their total from 3 wells in each treatment group was calculated and plotted.

### Cell cycle analysis.

Cell cycle assay was performed using the Cycletest Plus DNA Reagent Kit according to the manufacturer’s instructions (BD Biosciences) (55). Briefly, human glioma cell lines were seeded at a density of 1 × 10^5^ cells per 2 mL per well on 6-well plates with culture medium containing lomerizine (5 μM) or DMSO. After 24 hours of treatment, the cells were stained using the Cycletest Plus DNA Reagent Kit and were measured using a FACSCanto II system (Becton, Dickinson and Co.) and FlowJo software (Becton, Dickinson and Co.). The cells in the G_0_/G_1_, S, and G_2_/M phases were gated out as appropriate.

### Cell migration and invasion assays.

Cell migration and invasion assays were conducted using modified 24-well Boyden Transwell chambers separated by untreated membrane filter inserts (for migration measurements) or inserts precoated with Matrigel (for invasion assays) (BD Biosciences), as described previously (56, 57). Equal numbers of human glioma cell lines and differentiated GICs (dispersed from spheres as described above) were suspended in serum-free DMEM or DMEM/F12 and added to the upper chambers of each Transwell plate, whereas the lower chambers were filled with DMEM or DMEM/F12 supplemented with 10% FBS. Cells were treated with DMSO (vehicle) or lomerizine (1 μM or 5 μM). Equal concentrations were then added to the lower chambers. After 12 hours of incubation at 37°C, non-migrative/non-invasive cells were removed by wiping of the upper side of the filter, and the migrative/invasive cells in the lower chamber were fixed with methanol and stained using a Diff-Quik kit (Sysmex). The number of migrated/invaded cells was counted in 9 randomly chosen HPFs per treatment group, and the means from 3 wells were calculated. The relative values of the control group (0 μM) and each treatment group were then calculated and plotted.

### Western blot analysis.

Protein samples were extracted from the treated cells using a lysis buffer (Sigma-Aldrich) containing a mixture of protease and phosphatase inhibitors (Sigma-Aldrich). To determine the expression levels of the proteins of interest, whole-cell proteins were separated using gel electrophoresis, transferred to polyvinylidene fluoride membranes, and labeled with the indicated antibodies (Supplemental Table 1). The following primary antibodies were used in Figure 3: anti-CDK4 (Cell Signaling, 12790), anti-CDK6 (Cell Signaling, 13331), anti-PARP (Cell Signaling, 9542), and anti–cleaved PARP (Cell Signaling, 5625). The following primary antibodies were used in Figure 5 and Supplemental Figure 12: anti-SOX2 (GeneTex, GTX101507). The following primary antibodies were used in Figure 6 and Supplemental Figures 7, 8, and 11: anti-STAT3 (Cell Signaling, 12640), anti–p-STAT3 S727 (Cell Signaling, 9134), anti–p-STAT3 Y705 (Cell Signaling, 9145). The following primary antibodies were used in Figure 7 and Supplemental Figure 7: anti-Akt (Cell Signaling, 9272), anti–p-Akt (Cell Signaling, 4058), anti-ERK (Cell Signaling, 4695), anti–p-ERK (Cell Signaling, 4370). Anti–β-actin (FUJIFILM Wako Pure Chemical Corp., 010-27841) was used as a loading control in all western blots. Immunoblot signals were measured using a CS analyzer (version 2.0, ATTO).

### Tumor sphere-forming assay.

Briefly, the GIC spheres were dissociated into single cells using StemPro Accutase (Thermo Fisher Scientific). Cells were seeded on 96-well Costar ultra-low-attachment plates (Corning) at a density of 3.0 × 10^3^ cells per 200 μL of neurosphere medium supplemented with 1.0% methylcellulose. Cells were treated with either lomerizine (1, 5, or 10 μM) or DMSO for 7 days, and spheres larger than 100 μm in diameter were counted. The average number of spheres from the 6 wells in each treatment group was calculated and plotted.

### Limiting dilution assay.

Briefly, the GIC spheres were dissociated into single cells using StemPro Accutase (Thermo Fisher Scientific). GICs were seeded on 96-well Costar ultra-low-attachment plates (Corning) at 1, 5, 10, 25, 50, and 100 cells per well. Cells were treated with either lomerizine (1, 5, or 10 μM) or DMSO for 5 days, and the number of neurospheres in each well was quantified by manual counting. Data were quantified by Extreme Limiting Dilution Analysis (ELDA) software (http://bioinf.wehi.edu.au/software/elda/) (44).

### Target silencing by siRNA.

Two different sequences of siRNAs for STAT3 and negative control (QIAGEN) were used. Target sequence 1 of human STAT3 (siSTAT3-1) (SI00048377) was 5′-CTGGTCTTAACTCTGATTGTA-3′; target sequence 2 of human STAT3 (siSTAT3-2) (SI02662338) was 5′-CAGCCTCTCTGCGCAGAATTCAA-3′. Cells were seeded into 6-well flat-bottomed tissue culture plates at a density of 2 × 10^4^ cells per 2 mL DMEM for 24 hours. Subsequently, STAT3 siRNAs and control siRNA were transfected into DKGS01, DKGS10, and DKGS15 using Lipofectamine 2000 reagent (Thermo Fisher Scientific). Cells were incubated for 48 hours after transfection and used for the experiments. The downregulation of STAT3 was confirmed by Western blotting. To confirm the importance of STAT3 inhibition for lomerizine, Alamar blue assay using lomerizine, 2 different STAT3 siRNAs, and their combination was performed. The lomerizine-exposed groups were treated with 5μM lomerizine.

### Quantitative RT-PCR.

An RNeasy Mini Kit (QIAGEN) was used for total RNA extraction from cells and tissues. cDNA was synthesized using Transcriptor Universal cDNA Master (Roche Diagnostics) with 1 μg total RNA, and mRNA levels were quantified using qTOWER3G and MasterPLUS SYBR Green (Roche Diagnostics) with the Analytik Jena qPCRsoft 3.4 system. Primers and primer sequences for each gene are listed in Supplemental Table 2. Glyceraldehyde 3-phosphate dehydrogenase (GAPDH) genes were used as internal reference control. The 2^–ΔΔCt^ method was used to calculate the relative expression levels of genes, according to the cycle threshold values of the target mRNAs.

### Whole-cell patch-clamp recordings.

Whole-cell patch-clamp recordings were performed at room temperature from cultured GICs constituting spheroids that were firmly settled onto laminin-coated glass coverslips. The external solution contained (in mM): 150 NaCl, 4 KCl, 10 HEPES, 10 BaCl_2_, 2 MgCl_2_, and 10 glucose (pH 7.4, adjusted with NaOH). Borosilicate glass pipettes (5–8 MΩ) were filled with an internal solution containing (in mM): 86 CsCl, 20 TEA-Cl, 10 HEPES, 10 EGTA, 0.5 CaCl_2_, 4 MgCl_2_, 4 Na-ATP, 0.4 Na-GTP, 2 QX314, and 10 Tris-phosphocreatine (pH 7.3, adjusted with CsOH). An EPC10/2 amplifier combined with PATCHMASTER software (HEKA) was used to control membrane voltage and record membrane currents. Series resistance was routinely compensated by 70%–80%. Data were filtered at 3 kHz and digitized at 10 kHz. Current traces were corrected offline for leak and capacitive currents using the P/4 protocol and analyzed with Igor Pro 9.0 (WaveMetrics).

### Calcium imaging.

Cultured GICs were grown on laminin-coated glass coverslips in 6-well flat-bottomed tissue culture plates (Corning) at a density of 3.0 × 10^5^ cells per 1,000 μL for 2 hours. After the cells were washed with GIC culture medium, they were loaded with a calcium indicator (5 μM Fluo-4 AM; DOJINDO Laboratories) at room temperature for 30 minutes. After another 3 washes with GIC culture medium, time-lapse images were captured using an upright microscope (Olympus, Tokyo, Japan) and a water-¬ immersion objective (×40, NA 0.8; Olympus). Fluo-4 AM was excited with solid-state lighting (SOLA SM Light Engines, Lumencor) through an excitation filter (470–495 nm). Fluorescence images were detected by 10-millisecond exposures every 5 seconds through a dichroic mirror (505 nm) and an emission filter (510–550 nm) using a CMOS camera (Prime BSI Express, Teledyne Vision Solutions) and specific imaging software (MetaFluor version 7.7, Molecular Devices). Fluorescence signals were quantified by plotting of pixel intensities within an oval region of interest (ROI) encompassing each cell body. The baseline fluorescence (*F*_0_) for each ROI was defined as the average ROI intensity over the 10 frames preceding the event. The amplitude of the calcium fluorescence signal was calculated as Δ*F*/*F*_0_, where Δ*F* = *F* – *F*_0_. The T-type calcium channel activator SAK3 (1 nM) was administered 3 times. After application of lomerizine (5 μM), SAK3 was administered 3 additional times in the continued presence of lomerizine. Significant events were defined as fluorescence intensity changes exceeding 3 standard deviations of the baseline signal. Data were analyzed using GraphPad Prism 8 (Dotmatics).

### Xenograft glioma model treatment.

Following an institutional review board–approved protocol, we generated a mouse brain tumor model by transplanting KGS01 or KGS10 cells into the brains of 6-week-old female BALB/cSlc-nu/nu mice (Charles River Laboratories), according to our previous study (58). Briefly, a burr hole was drilled into the skull, 3 mm lateral to the bregma, and 1.0 × 10^6^ KGS01 or KGS10 cells were injected at a depth of 3 mm below the dura mater. The mice were randomly assigned to receive human-dose lomerizine, high-dose lomerizine, or 200 μL DMSO (equal volume vehicle) through daily oral administration (*n* = 6 mice per group). The human dose was the animal equivalent dose (AED) to that used clinically for migraine in Japan (20 mg/d) as calculated by the following formula: AED (mg/kg) = human dose (mg/kg) × (weight_human_ (kg)/weight_animal_ (kg)) ^(1^^−^^0.67)^ (59). Weight_human_ was set at 60 kg, the average for adults in Japan, and weight_animal_ at 0.02 kg, the average mouse body weight in this study cohort. The human-dose group was treated with 4.68 mg/kg of lomerizine. The dose for the high-dose group, 30 mg/kg, was defined based on previous reports indicating the absence of any adverse events when administered orally to mice, consistent with the present study on the neuroprotective effects of lomerizine (22). Treatment was initiated 2 days after transplantation and continued until sacrifice or death from GBM. After 55 days, all 18 mice were euthanized. The brain was excised, embedded in paraffin, cut into 4-μm serial coronal sections, and stained with hematoxylin and eosin. We calculated the surface included in the tumor contour of the ROI in the coronal sections showing the maximal area of each tumor. To estimate the overall survival time, 36 mice were randomly assigned to the same 3 treatment groups and individually monitored until death or for a duration of at least twice the mean survival of the control group. All animal experiments followed the Guidelines for the Care and Use of Laboratory Animals at Kanazawa University, which also conformed to national guidelines.

### Immunohistochemistry.

For immunohistochemistry, paraffin-embedded brain tissue blocks were sectioned at a thickness of 4 μm, mounted on slides, deparaffinized, autoclaved for 15 minutes in Target Retrieval Solution (TRS, Dako) and citrate buffer (pH 6.0) for antigen retrieval, incubated with methanol containing 3% hydrogen peroxide (H_2_O_2_) for 20 minutes to quench endogenous peroxidase activity, blocked for 20 minutes with 5% skim milk, and incubated with the indicated primary antibodies (Supplemental Table 1) or with nonimmune mouse or rabbit IgG as a negative control overnight at 4°C. The following primary antibodies were used in this study: anti–Ki-67 (Thermo Fisher Scientific, RM-9106), anti-nestin (BD Biosciences, 611658), and anti–p-STAT3 Y705 (Cell Signaling, 9145). After immunolabeling, the slices were washed and subjected to immunostaining by incubation with EnVision+ kit reagent (Dako) for 60 minutes at room temperature, followed by 3,3′-diaminobenzidine tetrahydrochloride treatment for 5 minutes. Sections were counterstained with hematoxylin and examined under a light microscope (Keyence). All images were acquired using a BZ-X700 microscope (Keyence) and digitally processed using the Keyence analysis software.

### Immunofluorescence.

For the immunofluorescence assay, the cells were fixed with 4% paraformaldehyde and permeabilized. After washing with PBS, the cells were blocked with 5% skim milk for 30 minutes. The slides were incubated overnight at 4°C with each antibody (Supplemental Table 1) and then incubated for 1 hour with Alexa Fluor 488 goat anti-rabbit antibody or Alexa Fluor 546 anti-mouse antibody at room temperature.

The following primary antibodies were used in this study: anti-CD133 (Abcam, ab19898), anti-CD44 (Cell Signaling, 3570), anti-GFAP (DAKO, Z0334), anti-nestin (BD Biosciences, 611658), anti-Olig2 (IBL, 18953), and anti-Tuj1 (R&D Systems, MAB1195).

Finally, the sections were mounted with DAPI-containing mounting medium (Santa Cruz Biotechnology) and photographed using a BZ-X700 microscope and analysis software (Keyence).

### Statistics.

Data are presented as mean ± SD. ANOVA and Tukey’s multiple-comparison tests were used to compare multiple groups, and 2-tailed unpaired Student’s *t* test was used to compare 2 groups in the mean or median values. Mouse survival was determined by construction of Kaplan-Meier curves, and group differences were evaluated using the log-rank test. A value of *P* < 0.05 was considered statistically significant for all tests. All statistical analyses were performed using Prism 8 software (Dotmatics).

### Study approval.

Experiments were designed and performed in strict accordance with the Ethical Guidelines for Medical and Health Research Involving Human Subjects (Ministry of Education, Culture, Sports, Science and Technology and Ministry of Health, Labor and Welfare) and were approved by the Ethics Committees of Kanazawa University (approval 209, 2080, and 3581). The animal experimental protocol was approved by the Research Ethics Committee of Kanazawa University, Kanazawa, Japan (approval AP-214260). Written informed consent was obtained from all the participants or their representatives.

### Data availability.

All data generated or analyzed in the article are included in the Supporting Data Values file.

## Author contributions

TI designed the experiments, conducted experiments, analyzed data, and wrote the original draft of the manuscript. S Tamai assisted with the experiments, discussed data, and edited the manuscript. NH assisted with the experiments. TM assisted with the experiments, discussed data, and edited the manuscript. KN assisted with the experiments. HS discussed data and edited the manuscript. S Tanaka, M Kinoshita, M Kobayashi, MM, and AH edited the manuscript. MN conceptualized and designed the experiments, interpreted data, and wrote and edited the manuscript.

## Conflict of interest

The authors have declared that no conflict of interest exists.

## Funding support

- Japan Society for the Promotion of Science, KAKENHI, 24K12239 (to TI).
- Fujita Memorial Fund for Medical Research (to TI).
- Kobayashi International Scholarship Foundation (to MN).
- Extramural Collaborative Research Grant of the Cancer Research Institute, Kanazawa University (to MN and AH).
- Intramural clinical research grant from Kanazawa University (to MN).

## Supplementary Material

Supplemental data

Unedited blot and gel images

Supporting data values

## Version 1 03/24/2026

In-Press Preview

## Version 2 05/08/2026

Electronic publication

## Version 3 08/18/2026

A typographical error was corrected in Supplemental Figure S7D
