# Supplementary material for: Repurposing T-type calcium channel blocker lomerizine as a therapeutic strategy for glioblastoma
Source: JCI Insight. 2026 Mar 24;11(9):e182522. doi: 10.1172/jci.insight.182522 (PMC13232010; doi:10.1172/jci.insight.182522)
Supplement: Unedited blot and gel images [file jciinsight-11-182522-s077.pdf]

Figure 2C

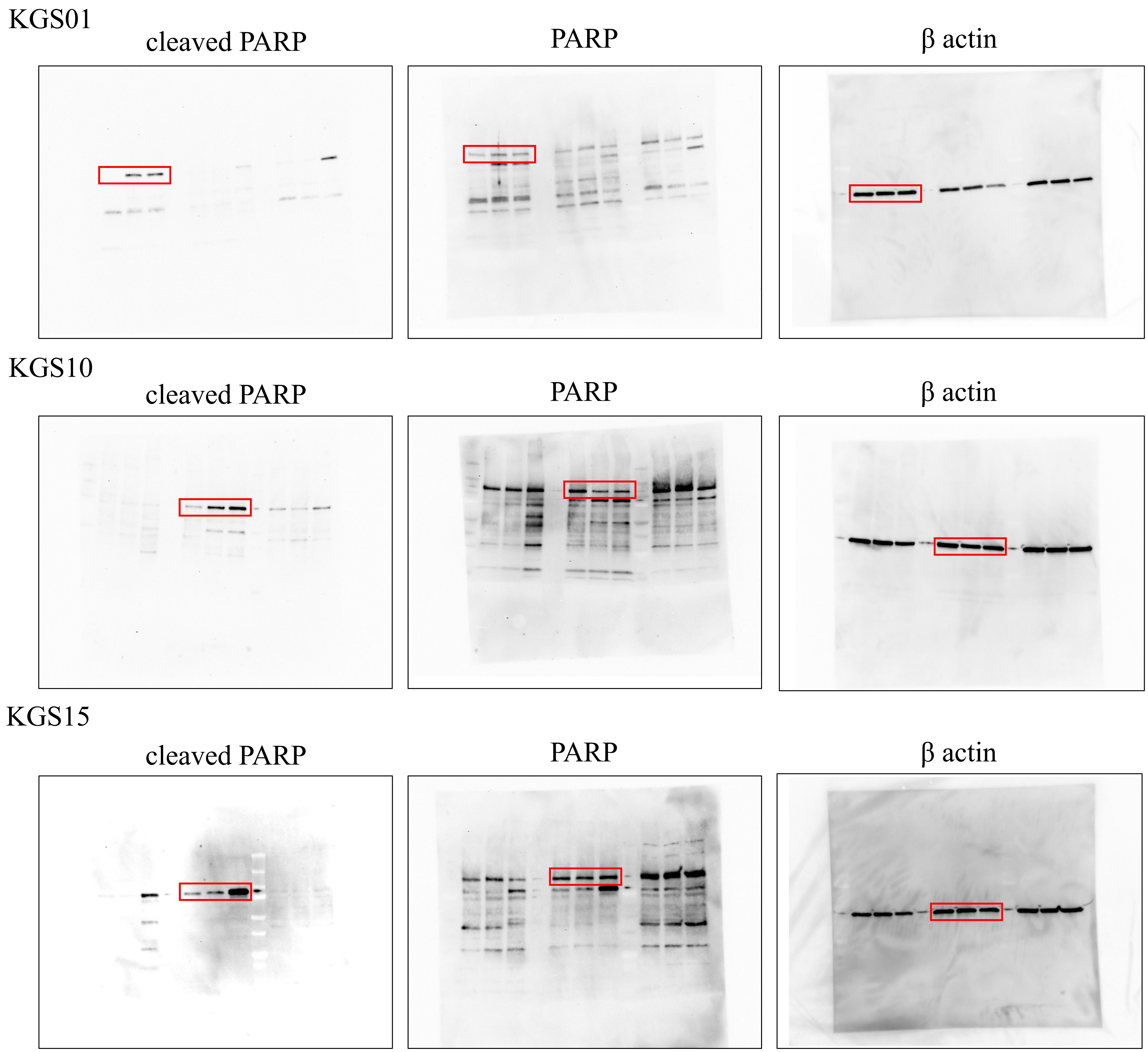
KGS01
cleaved PARP
PARP
β actin
KGS10
cleaved PARP
PARP
β actin
KGS15
cleaved PARP
PARP
β actin

Figure 2C

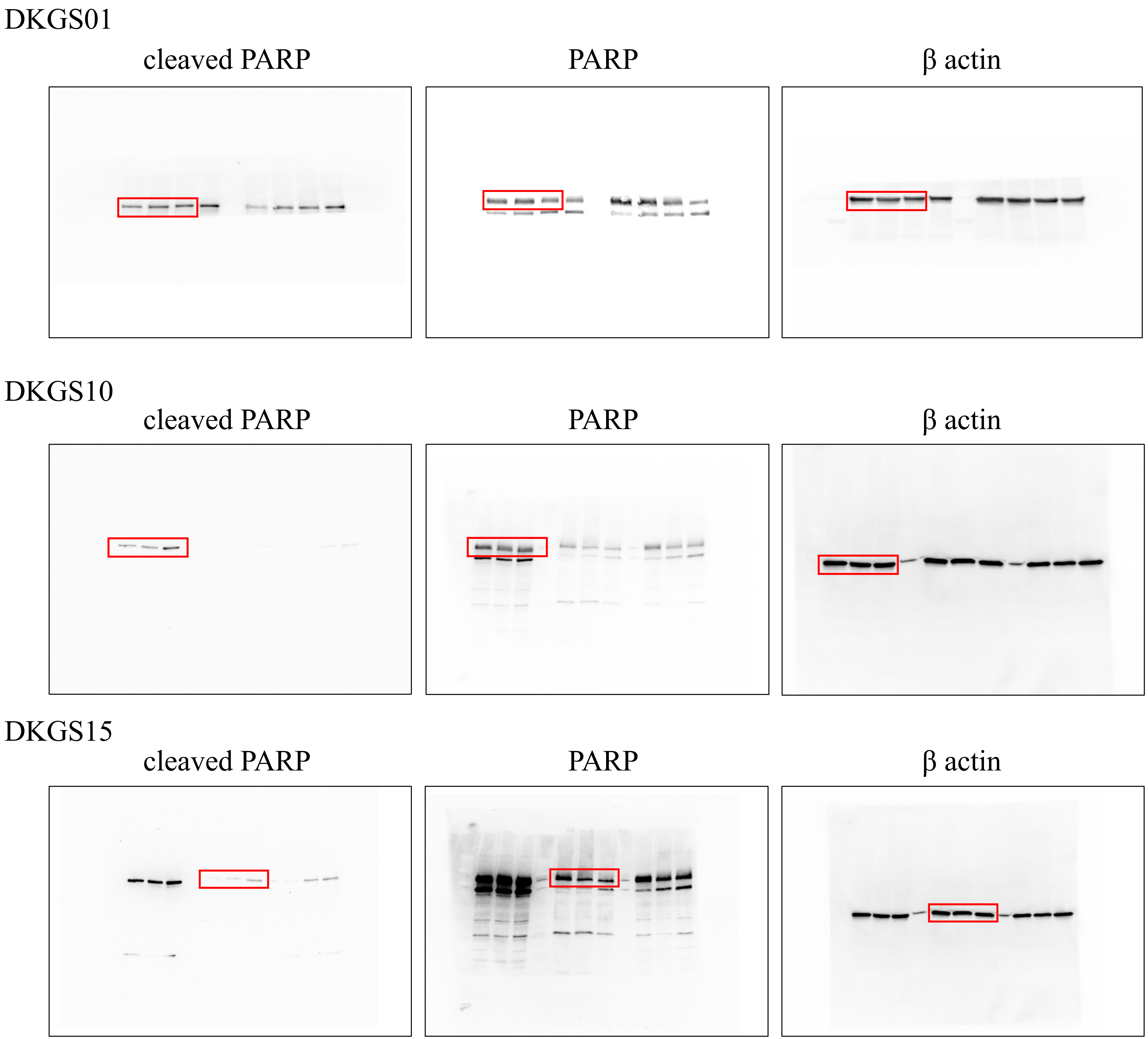
DKGS01
cleaved PARP
PARP
β actin
DKGS10
cleaved PARP
PARP
β actin
DKGS15
cleaved PARP
PARP
β actin

Figure 2C

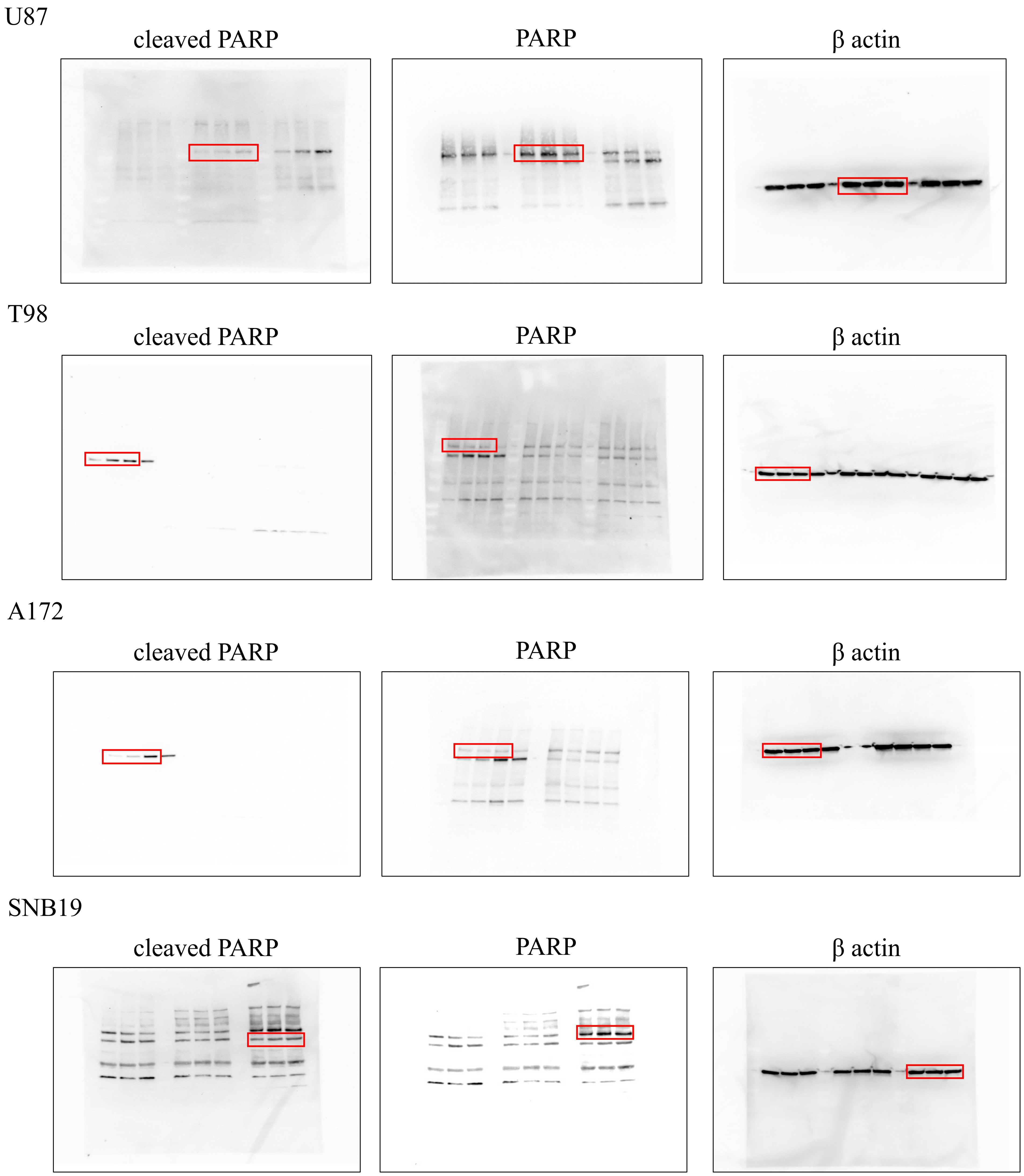
U87
cleaved PARP
PARP
β actin
T98
cleaved PARP
PARP
β actin
A172
cleaved PARP
PARP
β actin
SNB19
cleaved PARP
PARP
β actin

Figure 2E

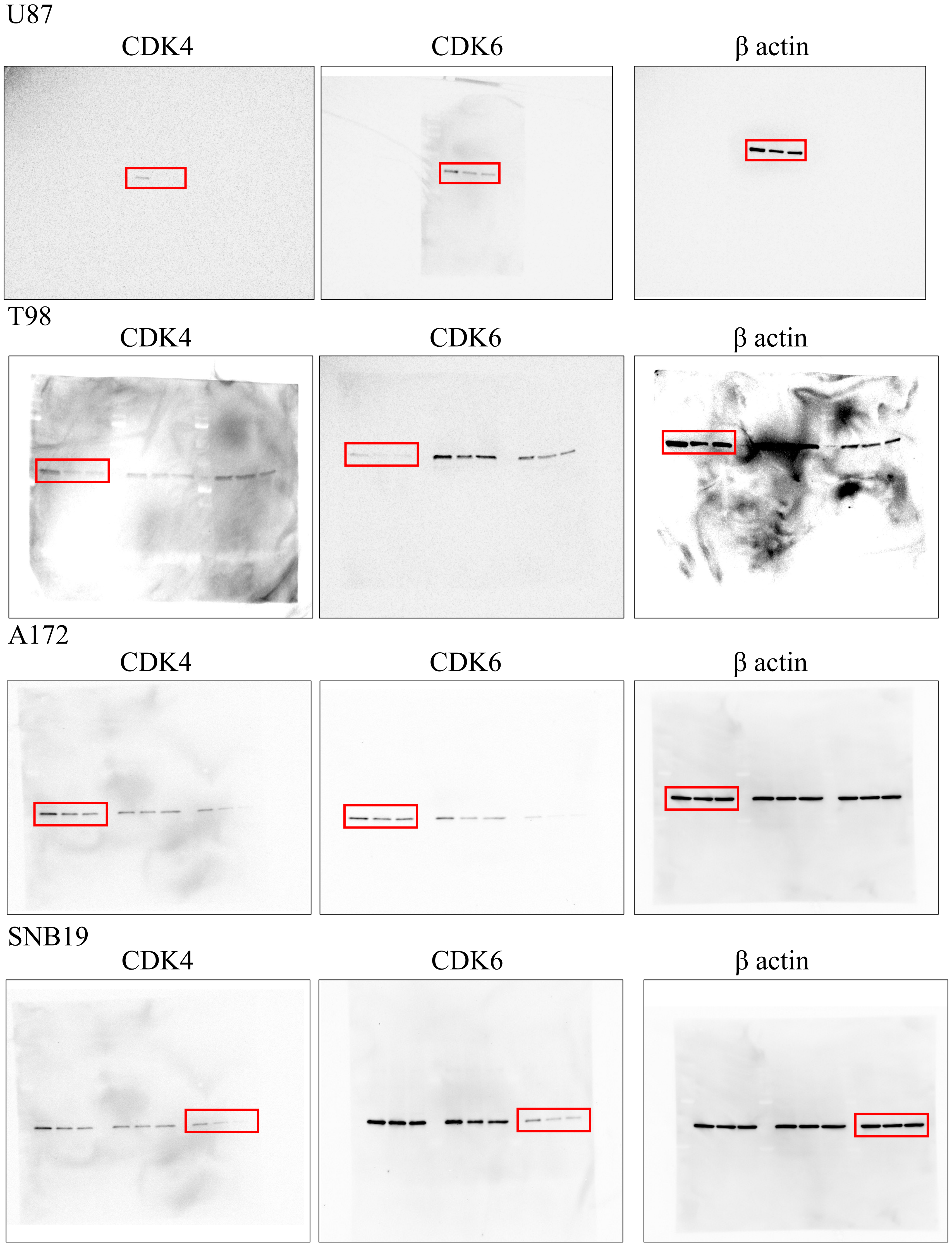
U87
CDK4
CDK6
β actin
T98
CDK4
CDK6
β actin
A172
CDK4
CDK6
β actin
SNB19
CDK4
CDK6
β actin

Figure 4D

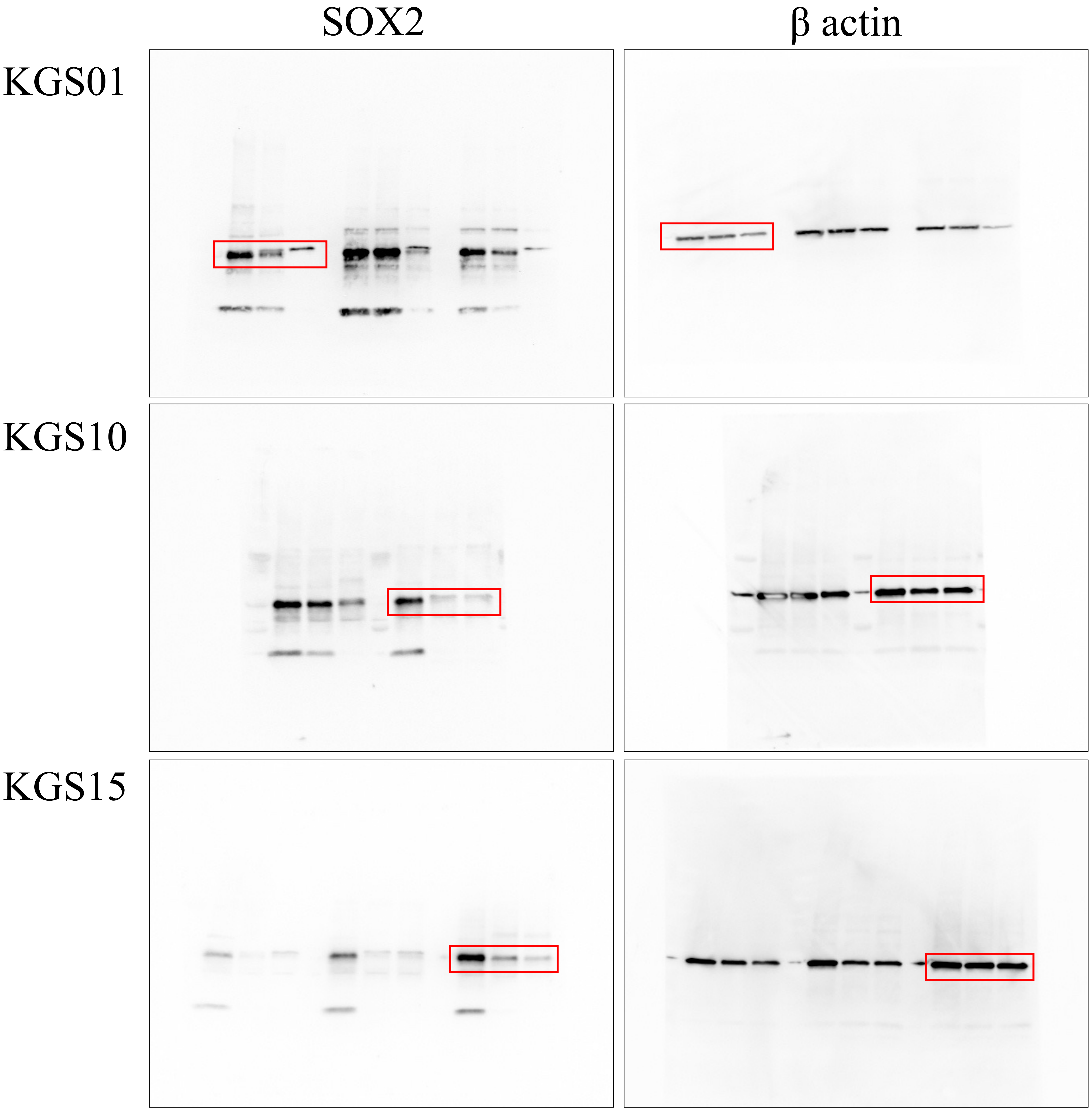

Figure 5A

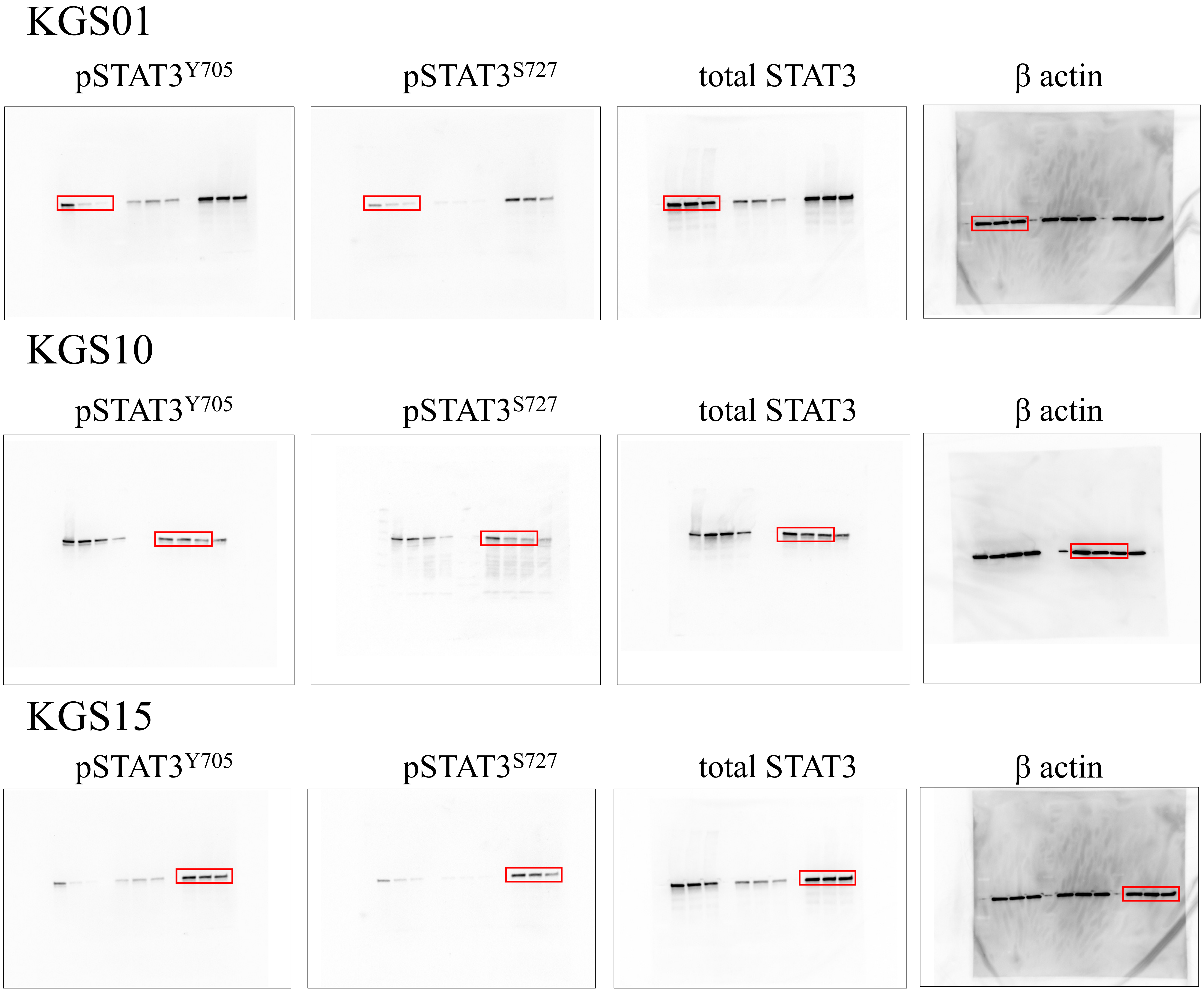
KGS01
pSTAT3Y705
pSTAT3S727
total STAT3
β actin
KGS10
pSTAT3Y705
pSTAT3S727
total STAT3
β actin
KGS15
pSTAT3Y705
pSTAT3S727
total STAT3
β actin

Figure 5A

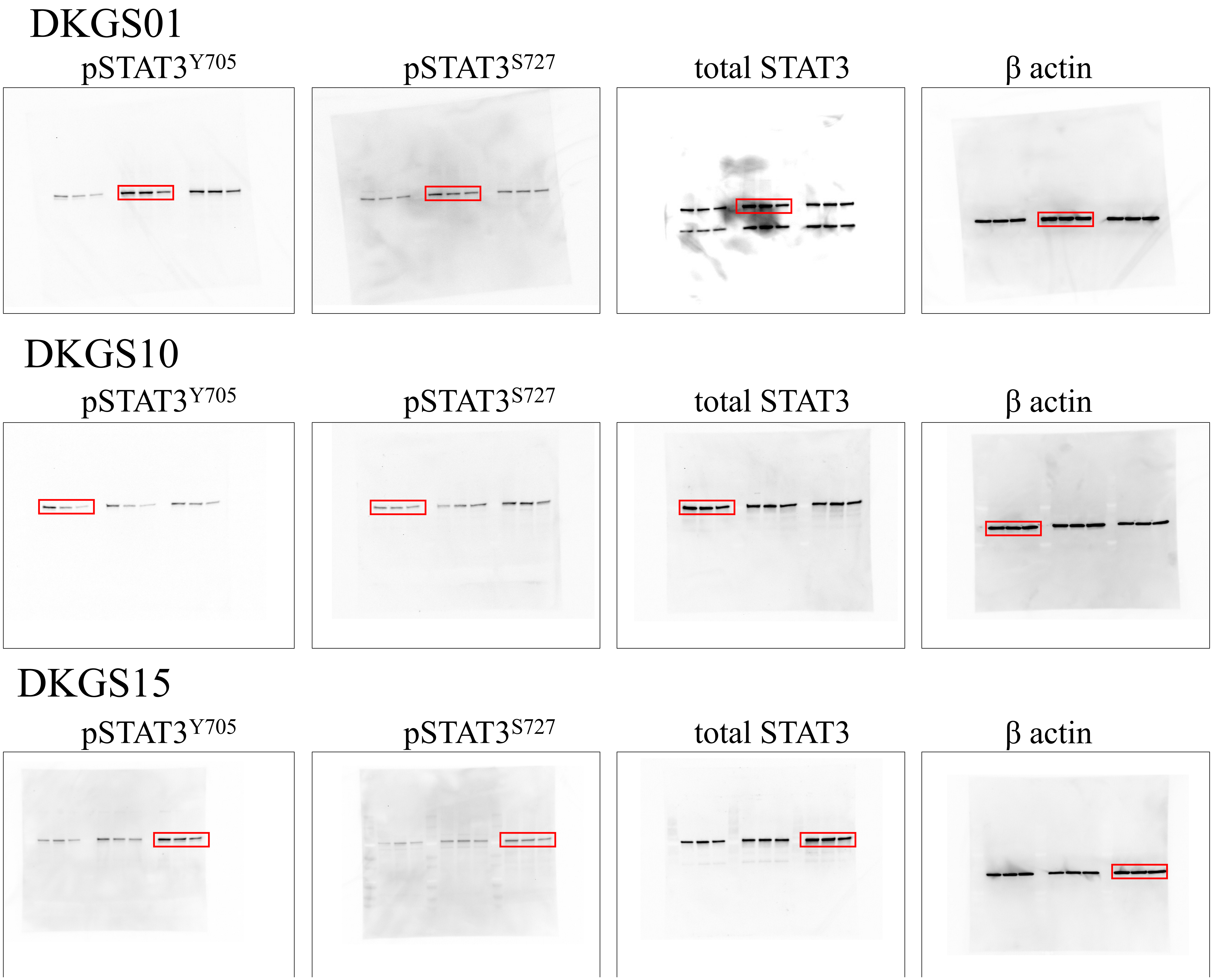
DKGS01
pSTAT3Y705
pSTAT3S727
total STAT3
β actin
DKGS10
pSTAT3Y705
pSTAT3S727
total STAT3
β actin
DKGS15
pSTAT3Y705
pSTAT3S727
total STAT3
β actin

Figure 5B

KGS01

pAKT AKT pERK ERK β actin

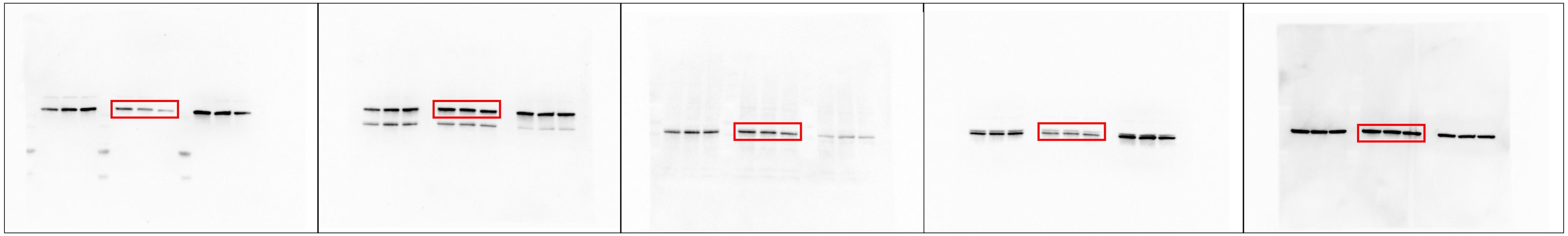

KGS10

pAKT AKT pERK ERK β actin

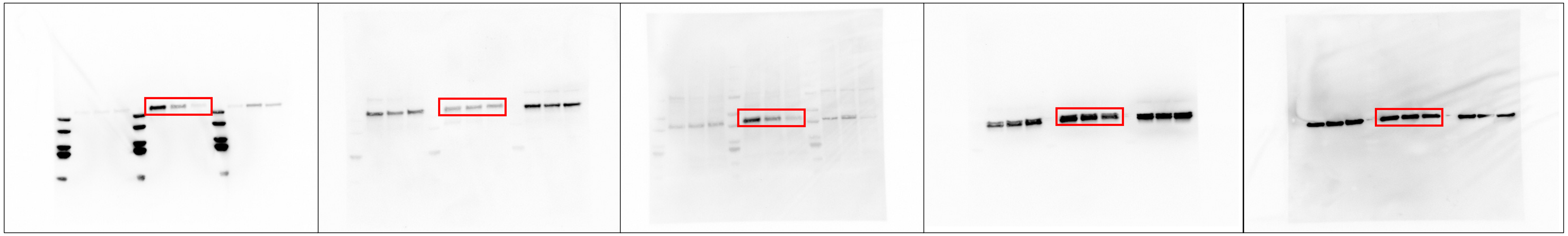

KGS15

pAKT AKT pERK ERK β actin

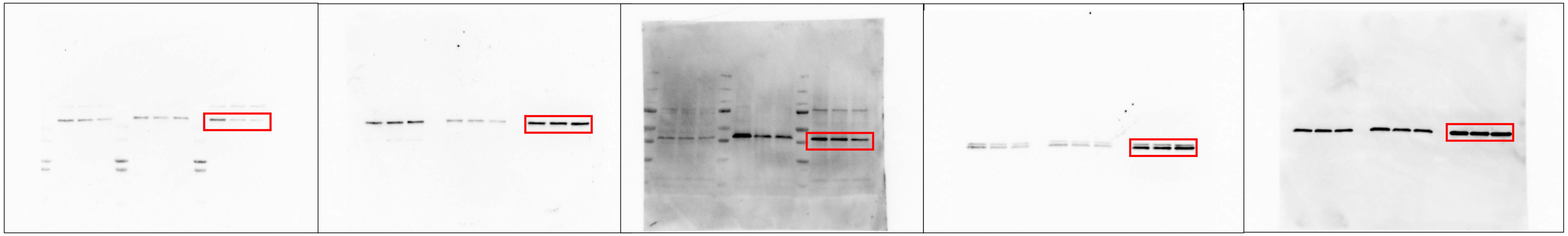

Figure 5B

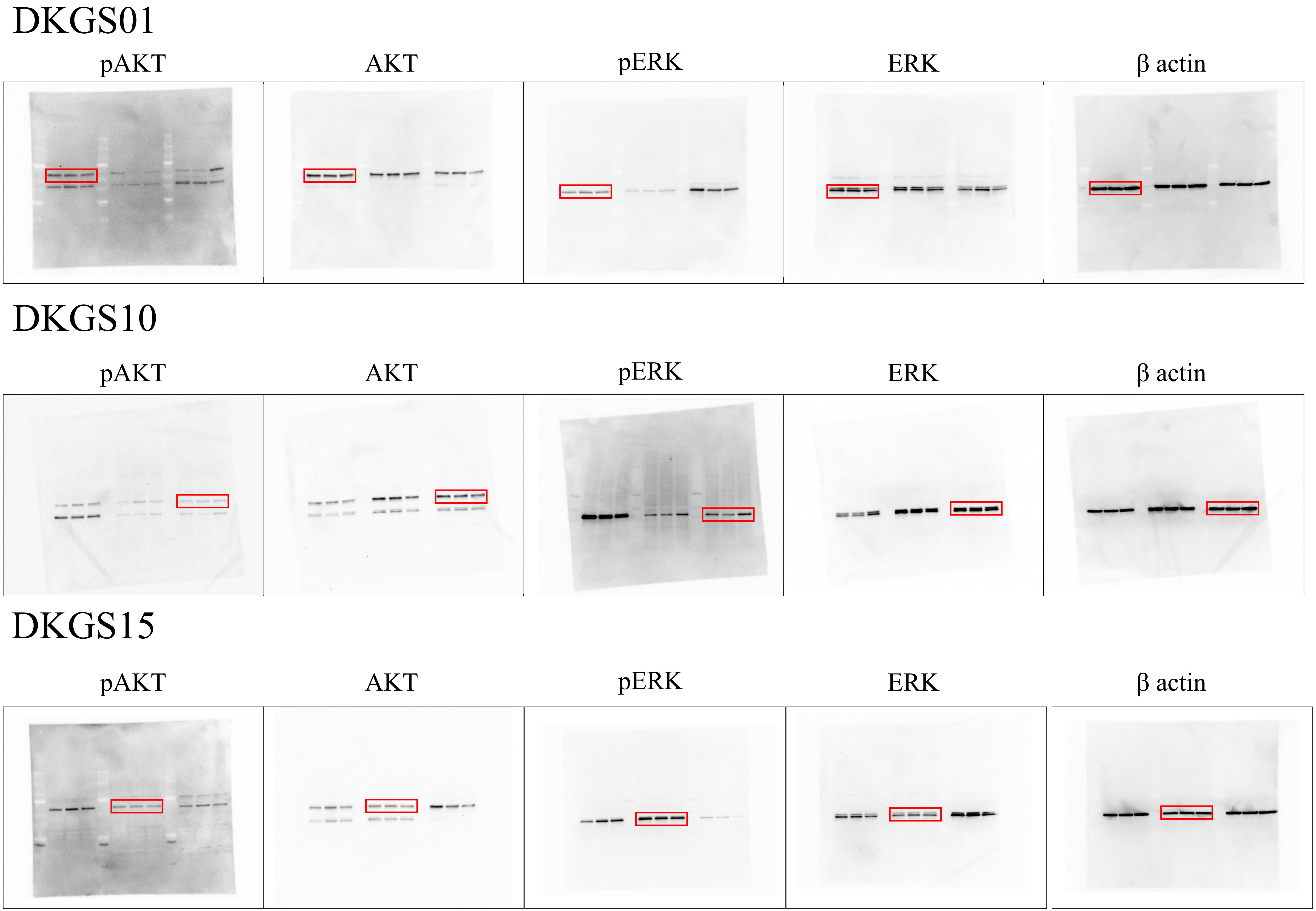
DKGS01
pAKT
AKT
pERK
ERK
β actin
DKGS10
pAKT
AKT
pERK
ERK
β actin
DKGS15
pAKT
AKT
pERK
ERK
β actin

Figure 5D

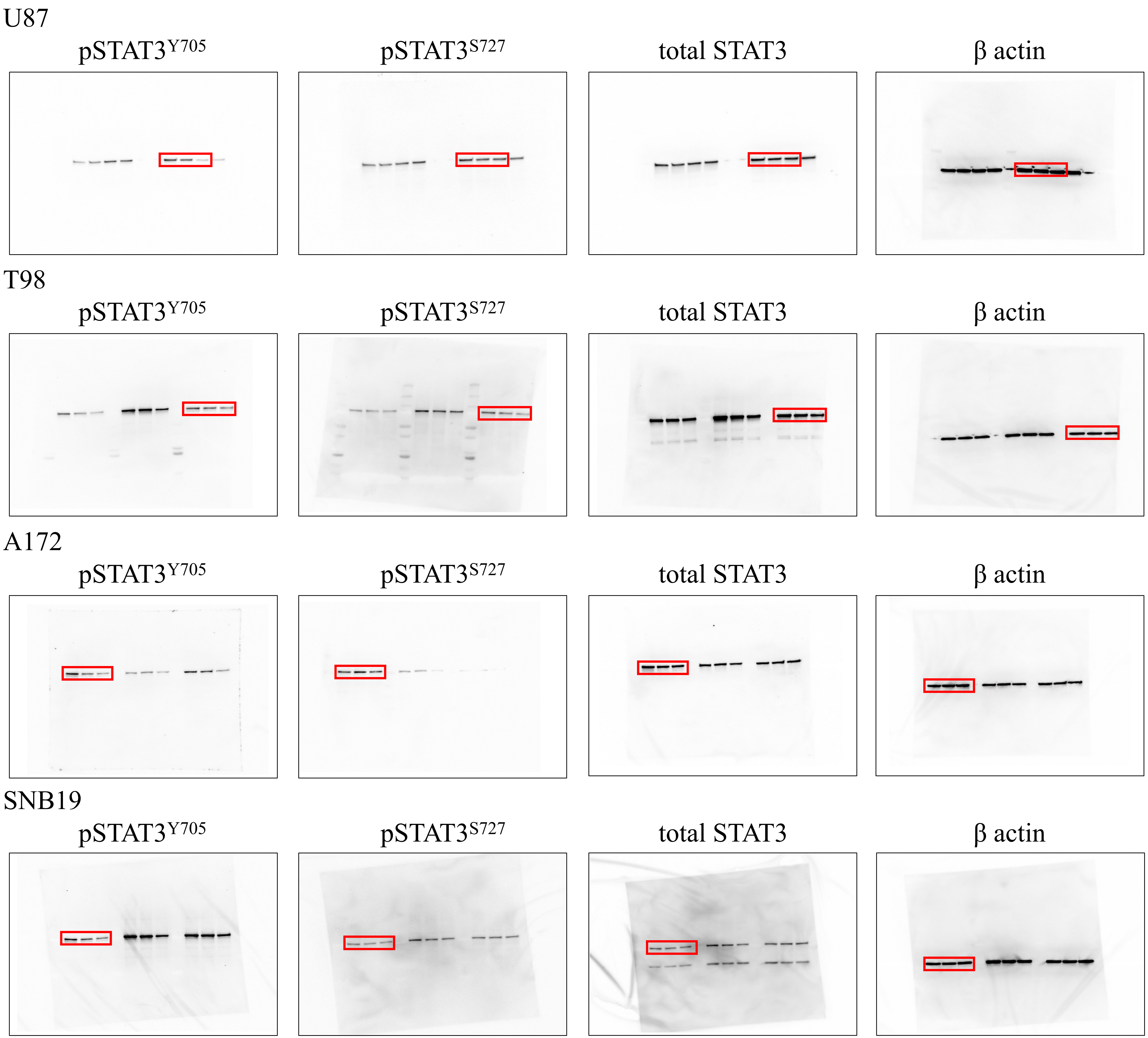

U87
pSTAT3Y705
pSTAT3S727
total STAT3
β actin
T98
pSTAT3Y705
pSTAT3S727
total STAT3
β actin
A172
pSTAT3Y705
pSTAT3S727
total STAT3
β actin
SNB19
pSTAT3Y705
pSTAT3S727
total STAT3
β actin

Figure 5E

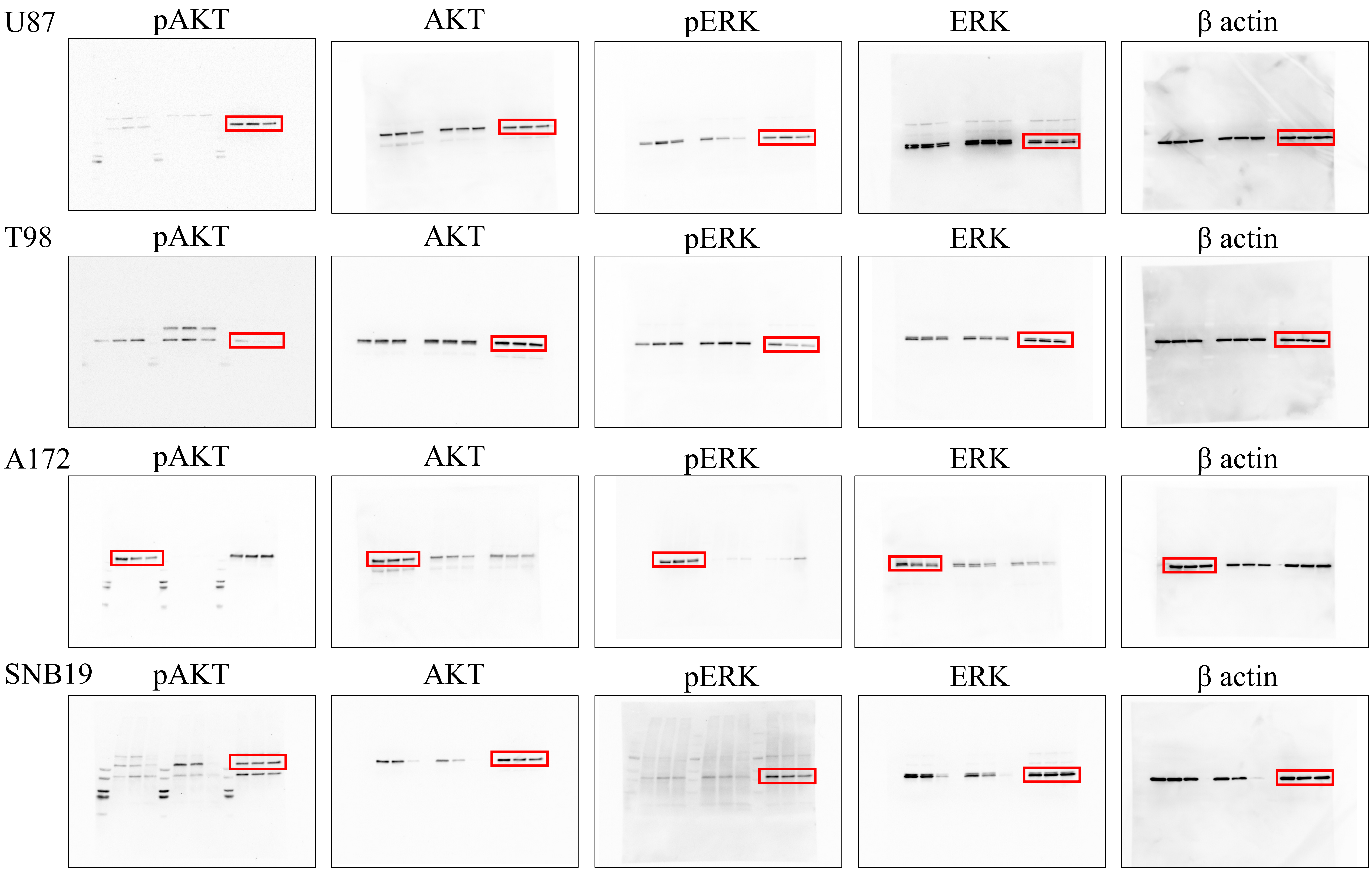
U87
pAKT
AKT
pERK
ERK
β actin
T98
pAKT
AKT
pERK
ERK
β actin
A172
pAKT
AKT
pERK
ERK
β actin
SNB19
pAKT
AKT
pERK
ERK
β actin
