# Supplementary material for: Repurposing T-type calcium channel blocker lomerizine as a therapeutic strategy for glioblastoma
Source: JCI Insight. 2026 Mar 24;11(9):e182522. doi: 10.1172/jci.insight.182522 (PMC13232010; doi:10.1172/jci.insight.182522)
Supplement: Supplementary file 3 [file jciinsight-11-182522-s010.pdf]

Supplementary Figure S1: Dose response curve used to generate IC50 for lomerizine

The 50% inhibitory concentration (IC50) of lomerizine in GICs (KGS01, KGS10, and KGS15) (**A**) and their differentiated cell lines (DKGS01, DKGS10, and DKGS15) at 72 h. The IC50 of GICs against lomerizine was significantly lower than that against their differentiated cells.

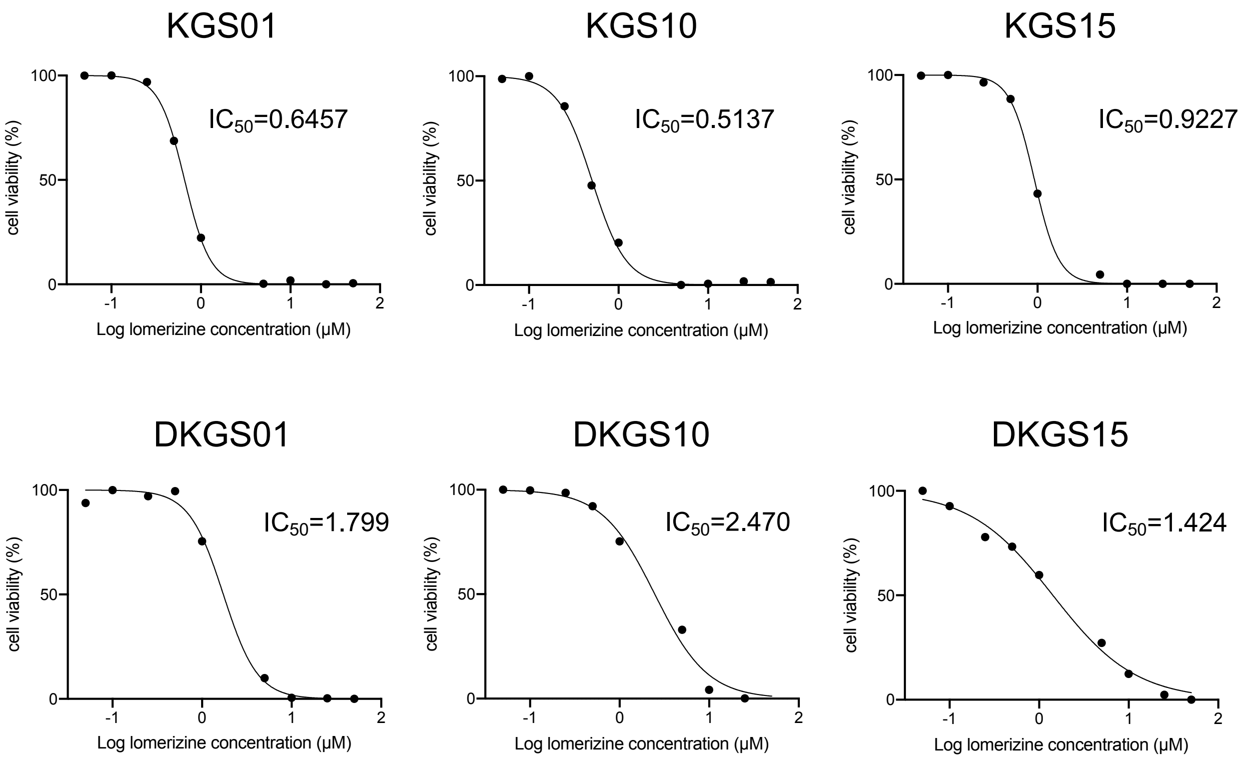

Supplementary Figure S2: Representative immunofluorescence images depicting apoptosis assay in glioma cell lines

The effect of lomeridine on the induction of apoptosis was evaluated using immunofluorescence, Annexin V assays, and western blotting *in vitro*. **A:** Representative images of nuclear staining of GIC cell lines (KGS01, KGS10, and KGS15) and their differentiated cells (DKGS01, DKGS10, and DKGS15) with or without lomerizine treatment for 24 h with Hoechst 33258 (left panel, blue), propidium iodide (PI) (middle panel, red), and their merged images (right panel). **B:** Representative images of nuclear staining of four glioma cell lines (U87, T98, A172, and SNB19) with or without lomerizine treatment for 24 h with Hoechst 33258 (left panel, blue), propidium iodide (PI) (middle panel, red), and their merged images (right panel). Indication of apoptotic cells is marked by condensed chromatin, with both Hoechst 33258- and PI-positive cells (pink). The enlarged image showing nuclear staining with PI and Hoechst 33258, with chromatin condensation in apoptotic cells. Bar graphs revealing the average number of double-stained apoptotic cells per high-power field for all cell lines. Scale bars: 100 µm. Data were analyzed by one-way ANOVA with Tukey's multiple comparisons test. *$p < 0.05$, **$p < 0.01$, ***$p < 0.005$ versus DMSO.

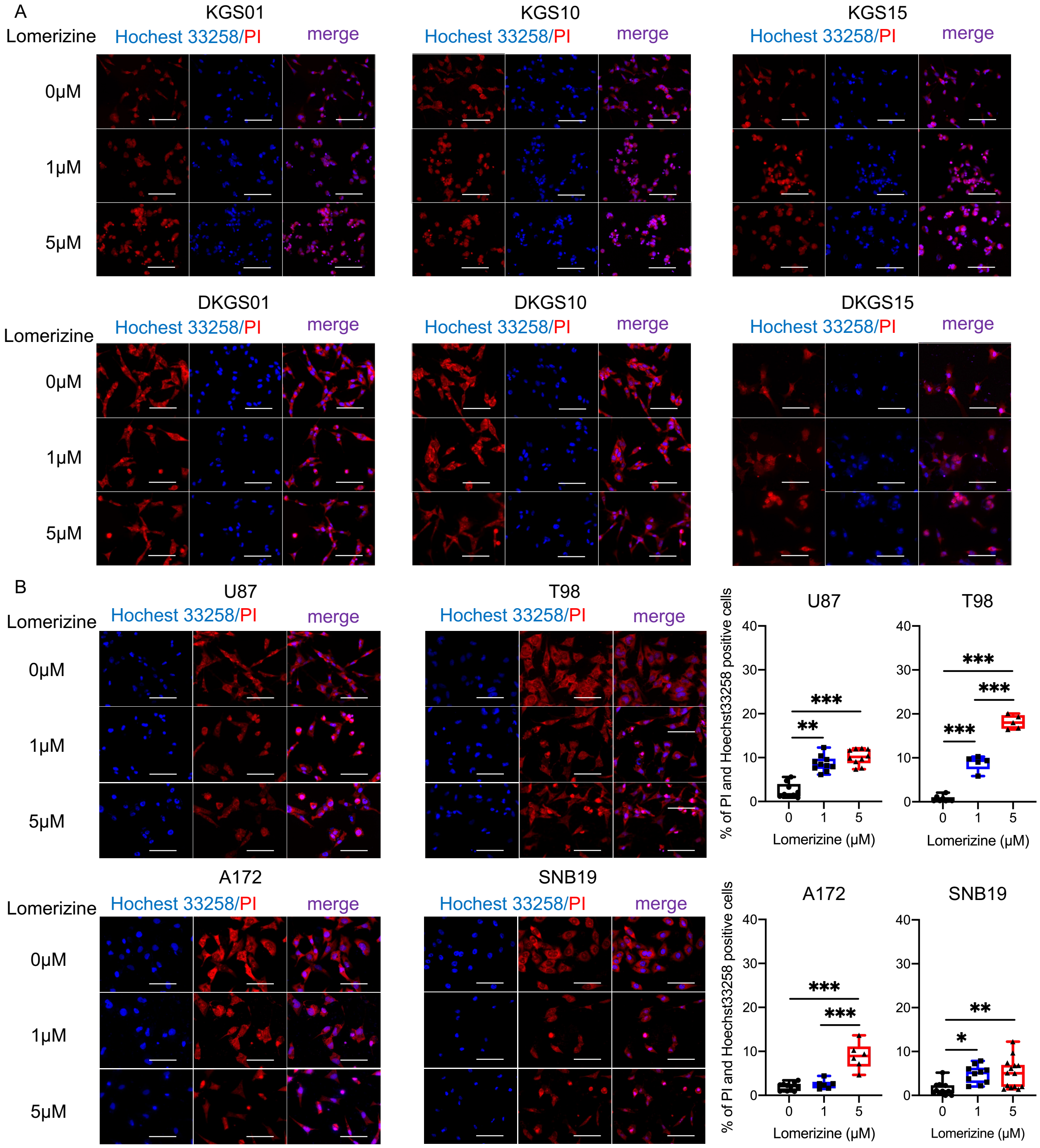

Supplementary Figure S3: Representative images of Annexin V/PI staining assays

Four human glioma cell lines (U87, T98, A172, and SNB19) were incubated with DMSO or lomerizine (1 μM or 5 μM) for 24 h and then analyzed for apoptosis using Annexin V/PI staining assays. Representative flow cytometry dot plots of apoptosis in four glioma cell lines. The histogram showing the percentages of early, late, and total apoptotic cells after treatment with different concentrations of lomerizine. The total number of apoptotic cells included both the early and late apoptotic cells. Data are presented as the mean ± standard error of triplicate experiments. Data were analyzed by one-way ANOVA with Tukey's multiple comparisons test. *$p < 0.05$, **$p < 0.01$, ***$p < 0.005$ versus DMSO.

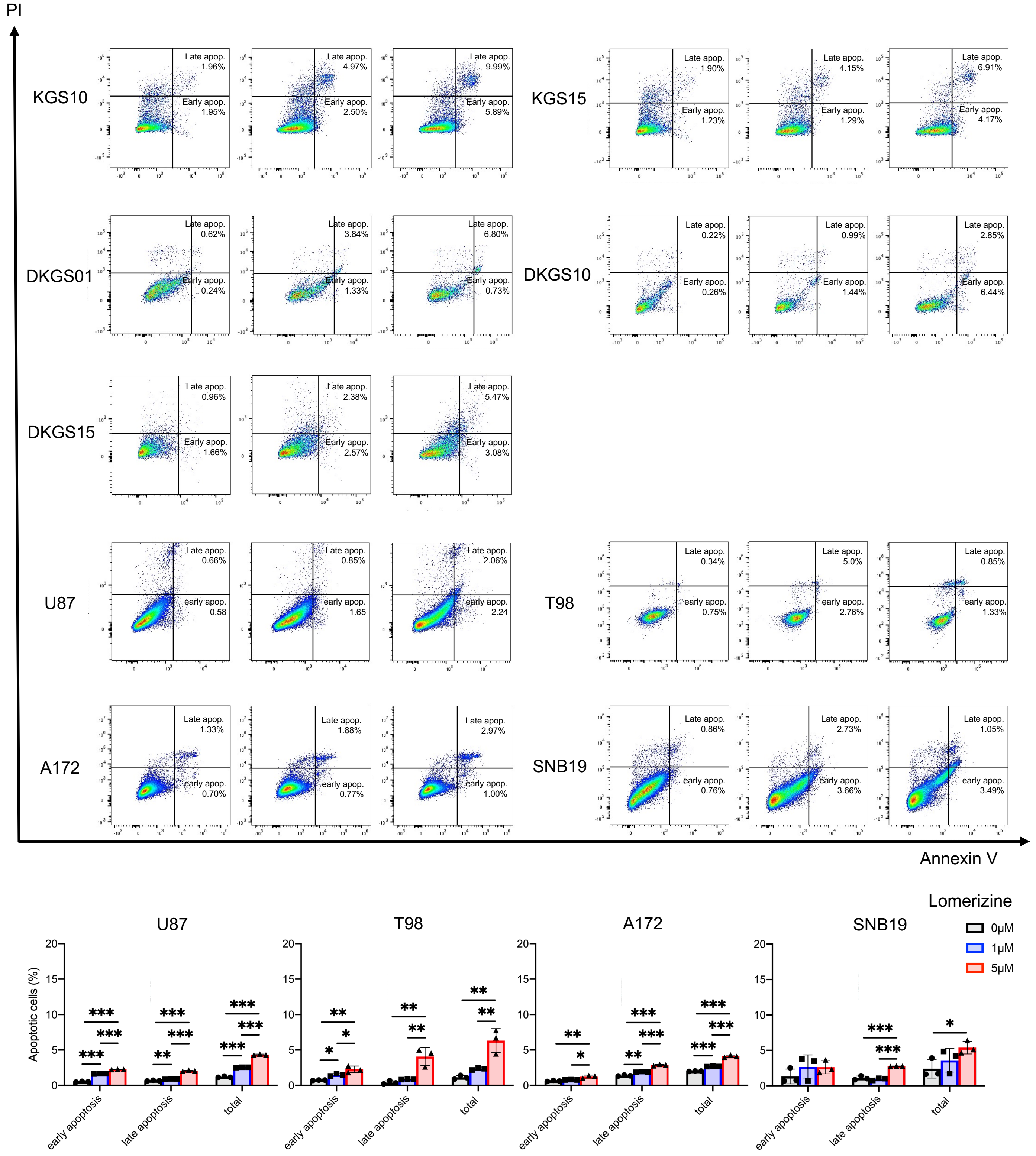

Supplementary Figure S4: Cell viability 12 hours after exposure to lomerizine

These graphs show the cell viability of three GICs (KGS01, KGS10 and KGS15) and their differentiated cells (DKGS01, DKGS10 and DKGS15) and four common GBM cell lines (U87, T98, A172 and SNB19) at 12 hours after lomerizine exposure. Data are presented as the mean ± standard error of triplicate experiments. Data were analyzed by one-way ANOVA with Tukey's multiple comparisons test. ns: not significant

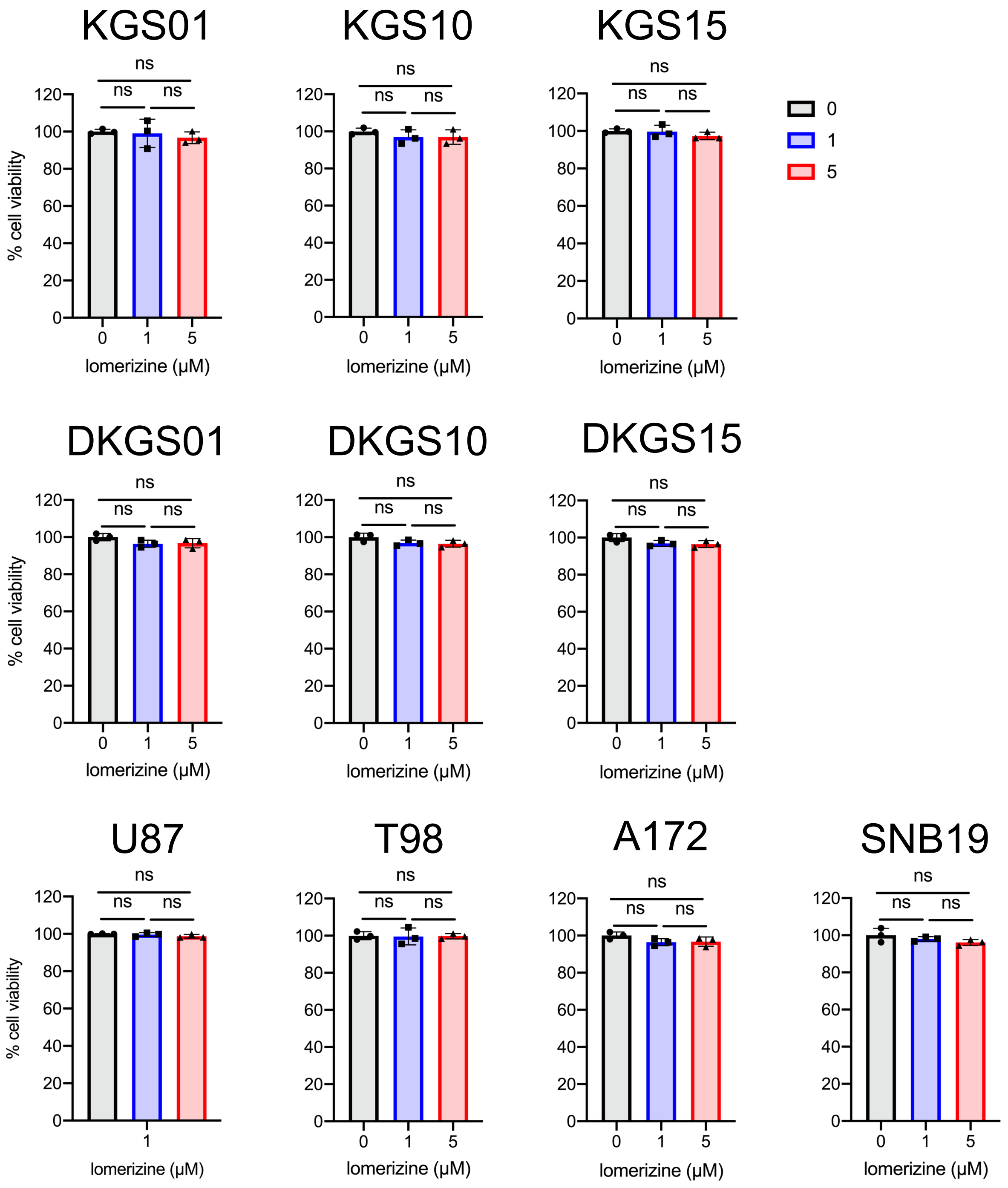

Supplementary Figure S5: Representative images of migration assay in glioma cell lines

Representative images of transwell migration assays of two GICs (KGS10 and KGS15), their differentiated cells and four human glioma cell lines (U87, T98, A172, and SNB19). **A, B:** Cells migrating through a non-Matrigel-coated transwell chamber were scored in the presence and absence of lomerizine for 12 h. Representative images of two GICs (KGS10 and KGS15), their differentiated cells and four human glioma cell lines Glioma cells were treated with lomerizine at 0 (DMSO), 1, and 5 µM concentrations. **C:** Cells invading through a Matrigel-coated transwell chamber were scored in the presence and absence of lomerizine for 12 h. Representative images of the transwell invasion assay of four glioma cell lines. The mean number of cells and the standard deviations were calculated for nine high-power microscopic fields **(C).** Data were analyzed by one-way ANOVA with Tukey's multiple comparisons test. $^{**}p < 0.01$, $^{***}p < 0.005$ versus DMSO.

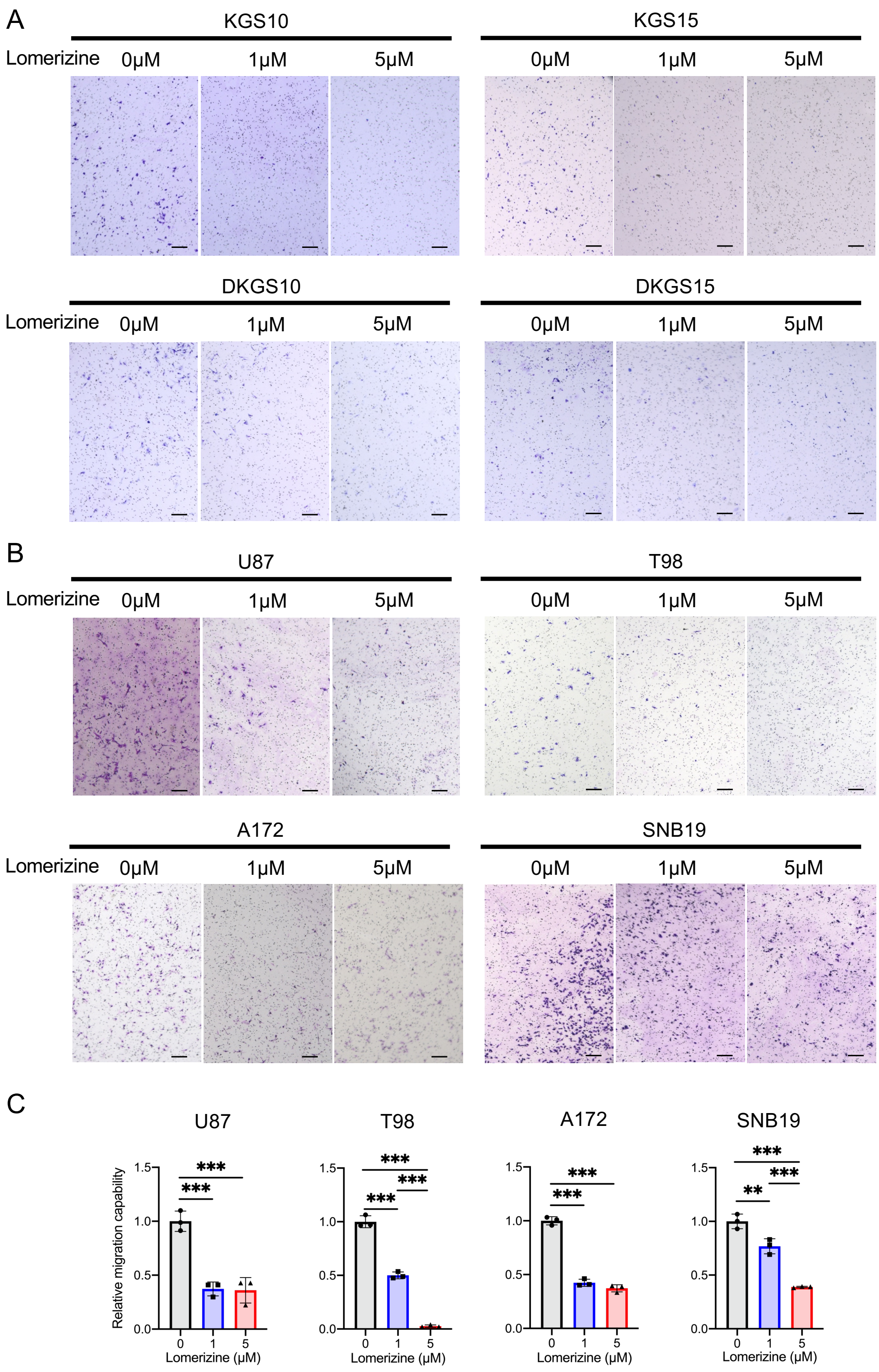

Supplementary Figure S6: Representative images of invasion assays in glioma cell lines

Representative images of transwell invasion assays of two GICs (KGS10 and KGS15), their differentiated cells and four human glioma cell lines (U87, T98, A172, and SNB19). **A, B:** Cells migrating through a non-Matrigel-coated transwell chamber were scored in the presence and absence of lomerizine for 12 h. Representative images of two GICs (KGS10 and KGS15), their differentiated cells and four glioma cell lines. Glioma cells were treated with lomerizine at 0 (DMSO), 1, and 5 µM concentrations. **C:** Cells invading through a Matrigel-coated transwell chamber were scored in the presence and absence of lomerizine for 12 h. Representative images of the transwell invasion assay of four glioma cell lines. The mean number of cells and the standard deviations were calculated for nine high-power microscopic fields **(C)**. Data were analyzed by one-way ANOVA with Tukey's multiple comparisons test. **p* < 0.05, ***p* < 0.01, ****p* < 0.005 versus DMSO.

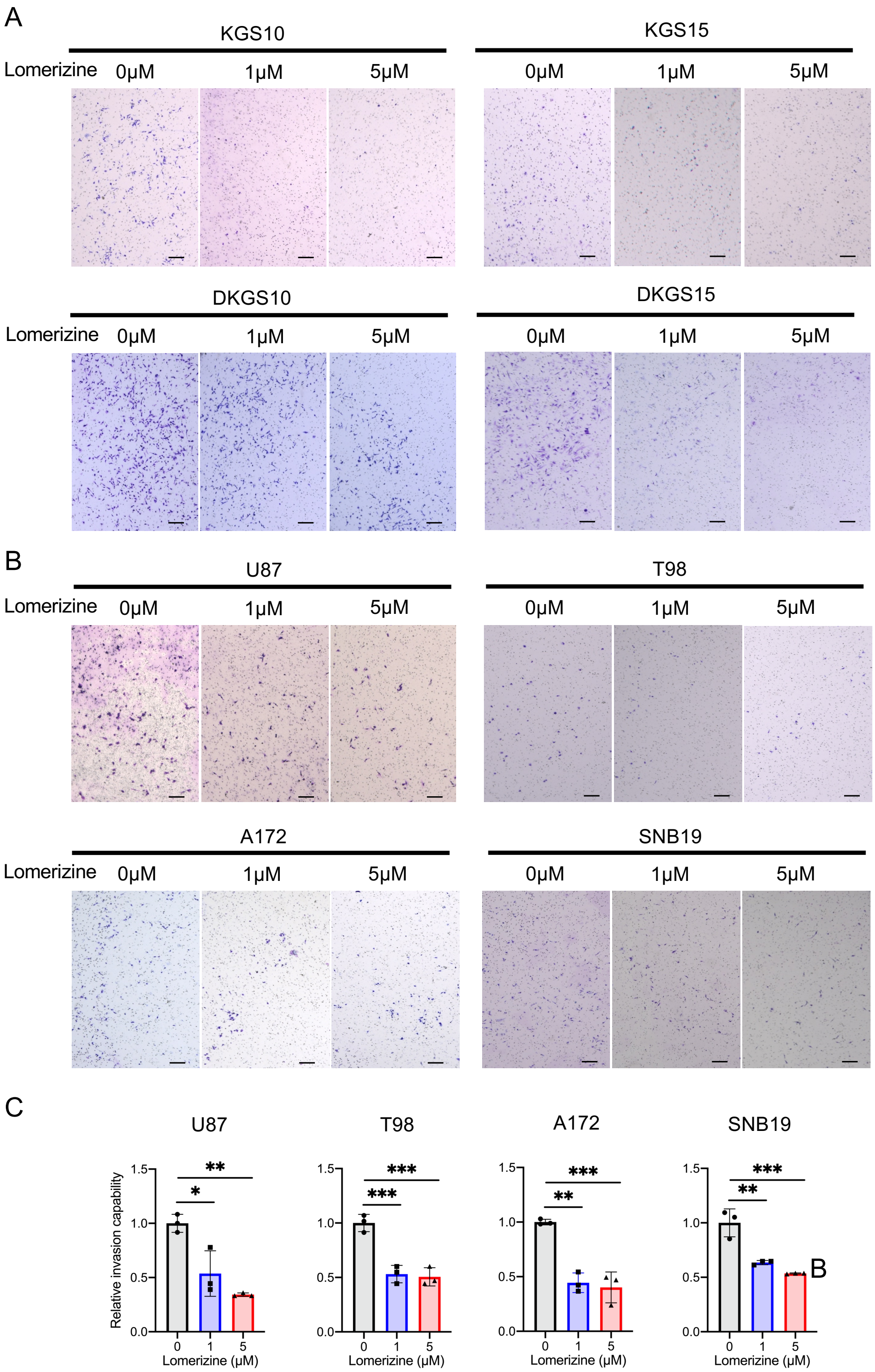

Supplementary Figure S7: Effects of lomerizine on cellular signaling pathway of glioblastoma cells.

Western blotting of three GICs (KGS01, KGS10, and KGS15) and their differentiated cells **(A-C)** and four human glioma cell lines (U87, T98, A172, and SNB19) **(D)** treated with lomerizine. All glioma cells were treated with lomerizine at 0 (DMSO) and 5 µM. Western blotting was performed on each cell line after 2h, 12 h and 24h of continuous exposure. The analyzed proteins included STAT3, AKT, ERK, phospho-STAT3^{Y705} (pSTAT3^{Y705}), phospho-AKT (pAKT), and phospho-ERK (pERK). The relative levels of protein expression were normalized to β-actin, serving as an internal control. **A:** Western blotting results for the expression of STAT3 and pSTAT3^{Y705} in the three GICs. **B:** Western blotting results for the expression of AKT, pAKT in the three GICs. **C:** Western blotting results for the expression of ERK, pERK in the three GICs. **D:** Western blotting results for the expression of STAT3, and pSTAT3^{Y705} in the four human glioma cell lines.

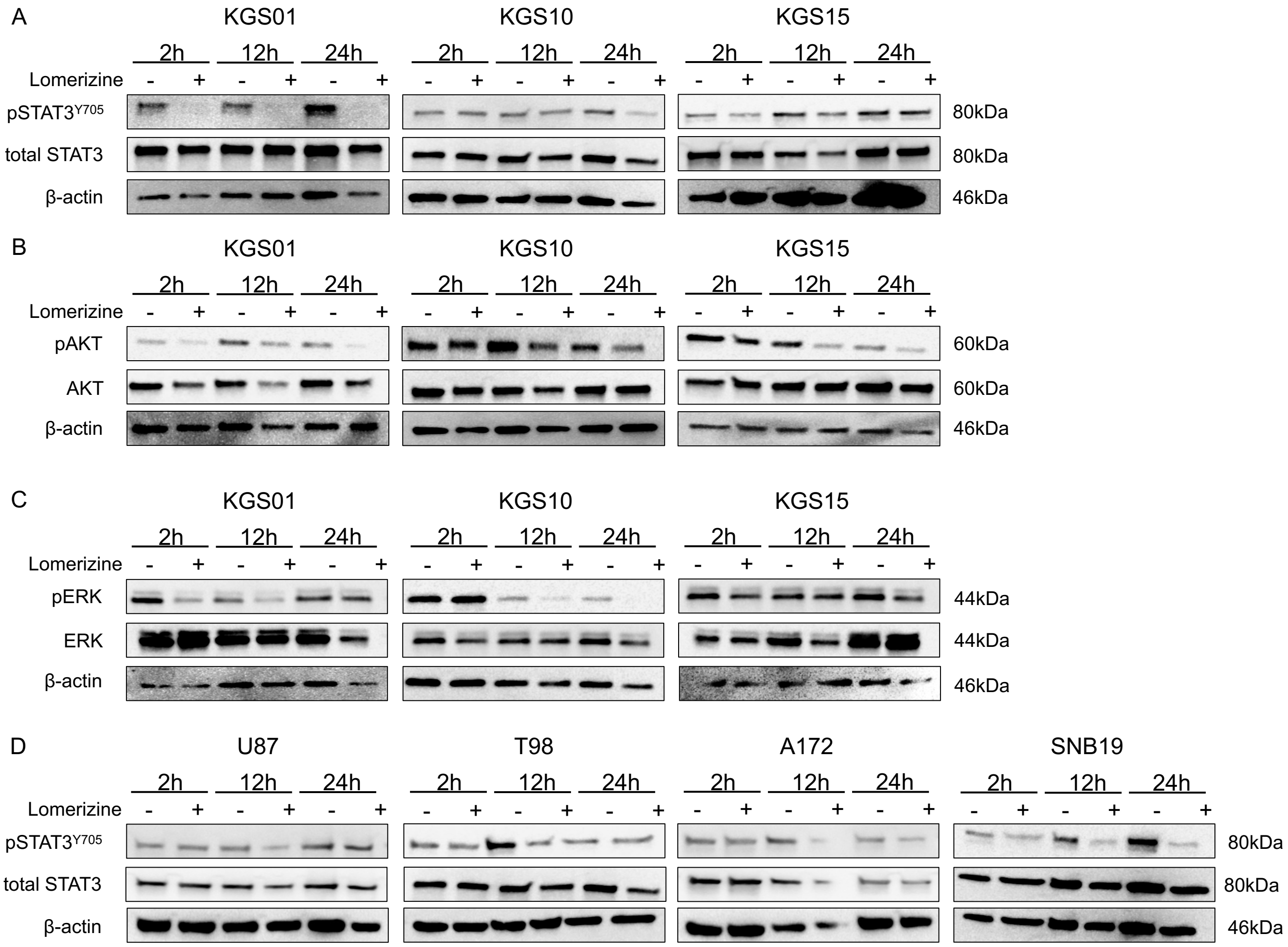

Supplementary Figure S8: Analysis with siRNA silencing STAT3.

A: Extracts of DKGS01, DKGS10 and DKGS15 cells treated by two different siRNA for STAT3 or negative control (NC) were immunoblotted with antibodies against STAT3 or β-actin. **B:** The proliferation of the GBM cells treated with lomerizine (lom) and si STAT3-1 and -2 was significantly reduced compared to GBM cells treated with si NC, whereas a little difference was found between lomerizine treated group and the combination of STAT3 siRNA and lomerizine.

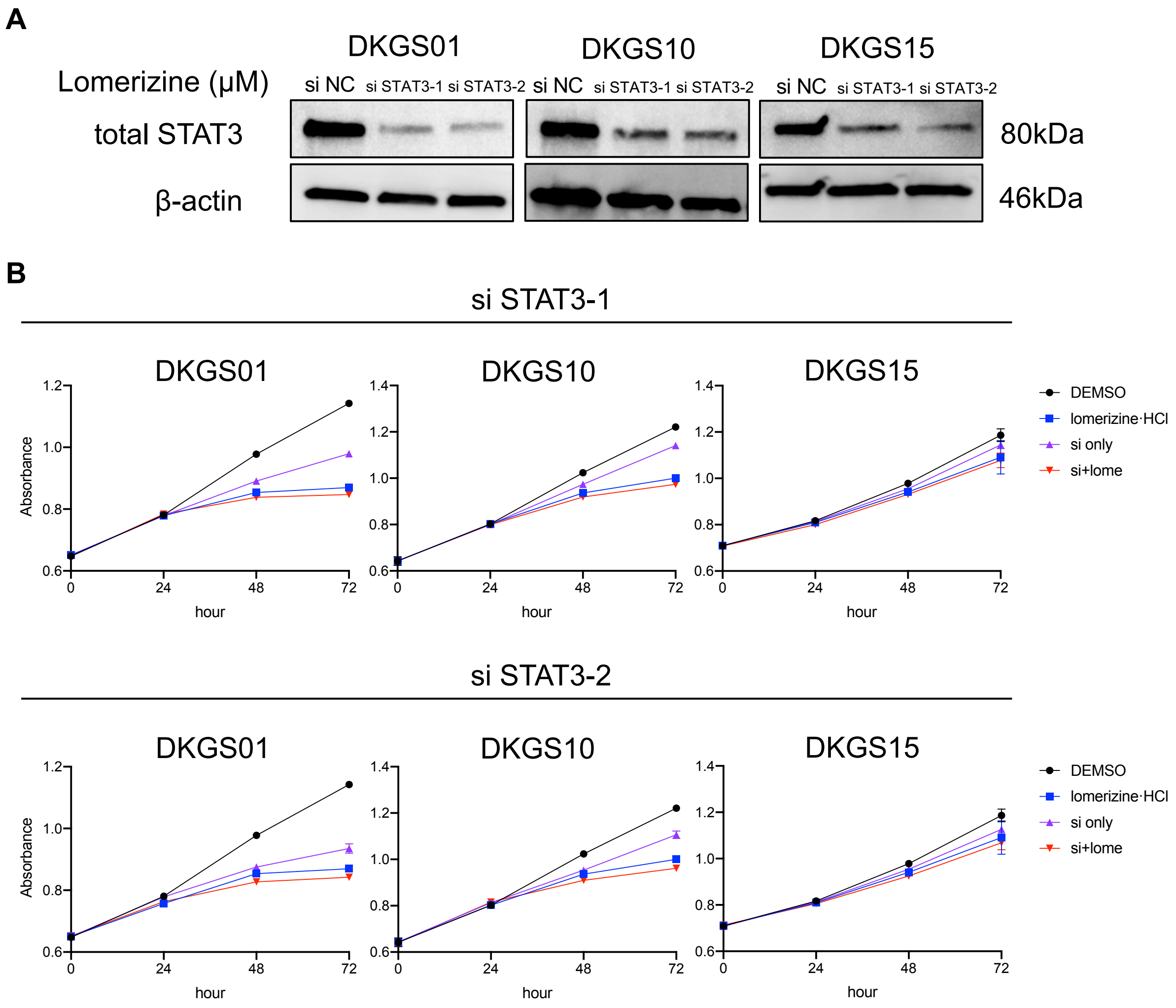

Supplementary Figure S9: Effects of lomerizine for normal tissues *in vivo*

A: Representative images showing hematoxylin and eosin staining of four organs (skin, liver, kidneys, and lungs) in a xenograft mouse model with or without high dose lomerizine treatment (30mg/kg). Scale bars: 100 µm. **B:** HFF-1, normal human fibroblasts, were treated with lomerizine at 0, 1, 5, 10 and 20 µM, and evaluated their proliferation by the percentage of AlamarBlue reduction. Results are shown as the mean ± SD of six independent experiments **(B)**. Data were analyzed by one-way ANOVA with Tukey's multiple comparisons test. ns: not significant

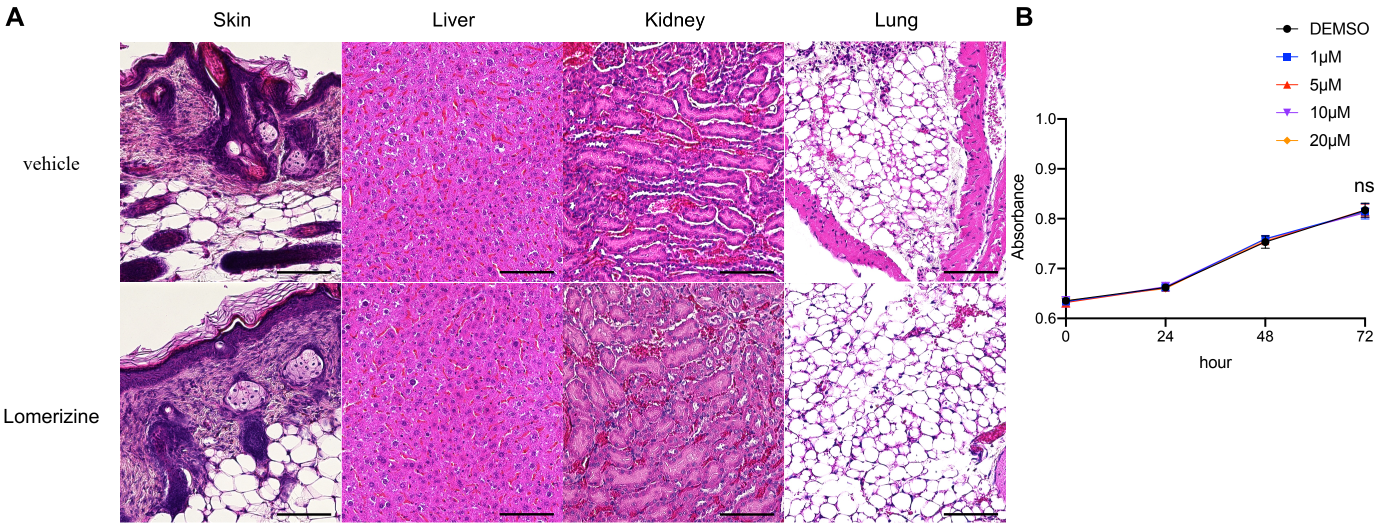

Supplementary Figure S10: T-type calcium channel activation in glioma-initiating cells and effects of lomerizine on T-type calcium channel.

(A): Quantitative rt-PCR results comparing stem cells and their differentiated cells for the expression of CACNA1G, CACNA1H, and CACNA1I). **(B, C)** T-type voltage-gated Ca^{2+} channel currents in GICs. **B**. Representative whole-cell currents recorded from a KGS01 cell in response to a 300-ms depolarizing step to –30 mV from holding potentials of –90 mV or –60 mV with 10 mM Ba^{2+} as the charge carrier. **C.** Current-voltage relationship obtained from peak currents elicited by a series of depolarizing pulses from a holding potential of –90 mV to test potentials ranging from –80 mV to 10 mV in KGS01 (white circle, n=5) and KGS10 cells (black circle, n=8). Plots are presented as mean ± SEM. **(D, E)** Calcium imaging showing that lomerizine suppresses SAK3-induced calcium elevation in GICs. **D.** Representative traces of SAK3-induced changes in Fluo-4 fluorescence in GIC (KGS10) and their inhibition by lomerizine. **E.** Peak $\Delta F/F_0$ of calcium responses in three GIC lines following application of the T-type calcium channel activator SAK3 (1 nM), with or without lomerizine (5 μM). Error bars represent the SD for each protein in individual cell groups from three separate experiments (**A and E**). Data were analyzed by two-tailed Student's *t* test. **A:** $*p < 0.05$, $**p < 0.01$, $***p < 0.005$ versus DMSO. **E:** $***p < 0.005$ versus control.

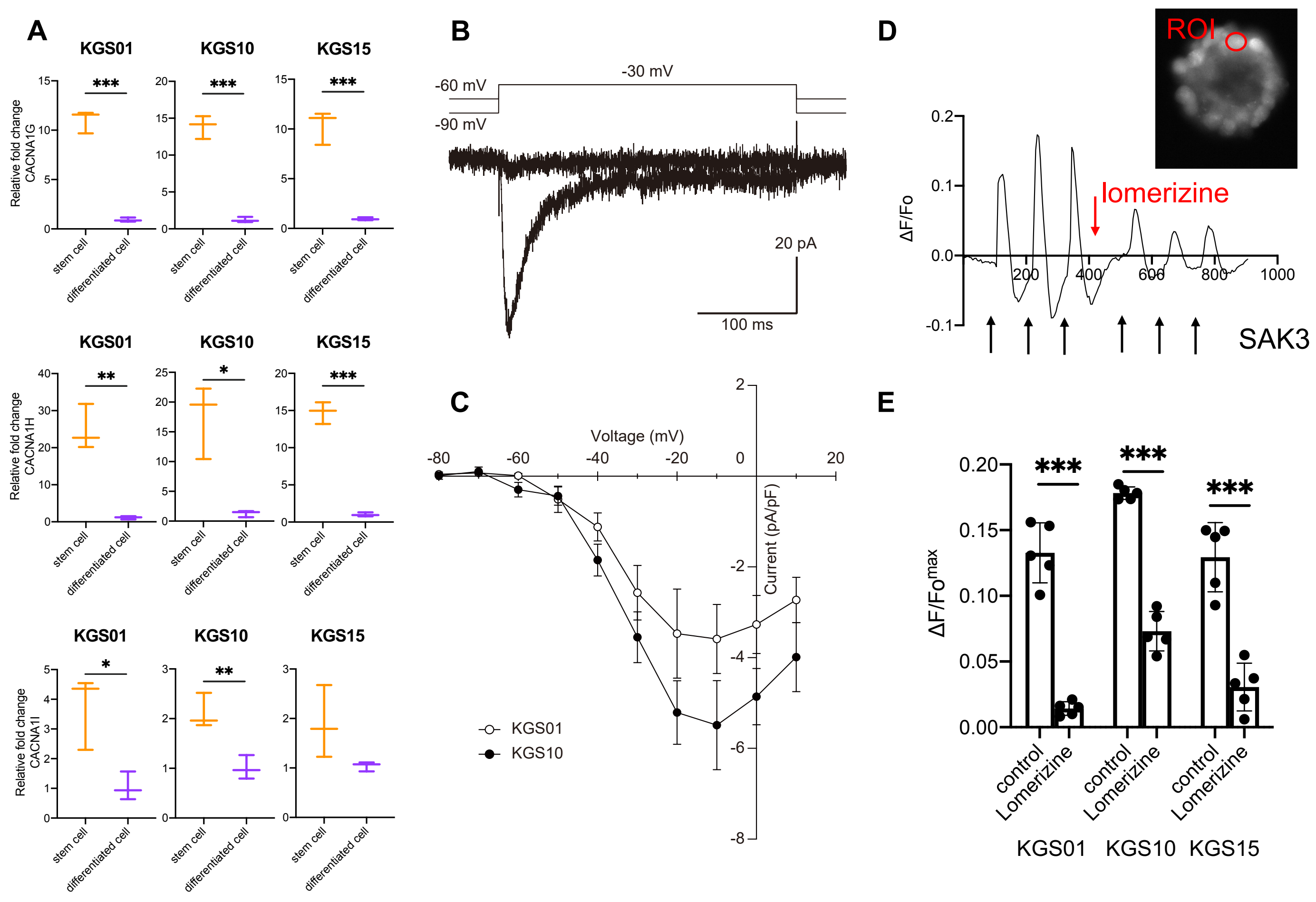

Supplementary Figure S11: Effects of lomerizine on STAT3 activation partially depends on the inhibition of T-type calcium channel activity.

A and B: AlamarBlue proliferation assays were performed using lomerizine (5 μM) and SAK3 **(A)** and ZSET1446 **(B)**, a potent enhancer of T-type calcium channels, in combination with three GICs and four common glioma cell lines and each growth curve was analyzed. Plates were read using a microplate reader at 24, 48, and 72 h. The cell proliferation inhibitory effect of lomerizine was suppressed by T-type calcium channel enhancer drugs in all GICs and glioma cell lines. **C and D:** Western blotting of three GICs (KGS01, KGS10, and KGS15) **(C)** and four human glioma cell lines (U87, T98, A172, and SNB19) **(D)** treated with/without lomerizine (5 μM) and SAK3 **(C)** and ZSET1446 **(D)**. The expression of phospho-STAT3 Y705 suppressed by lomerizine treatment was partially canceled by T-type calcium channel enhancer pretreatment in almost all GIC and glioma cell lines. Results are shown as the mean ± SD of six independent experiments **(A and B)**. Data were analyzed by two-tailed Student's t test. ns: no significant, *$p < 0.05$, **$p < 0.01$, ***$p < 0.005$ versus DMSO.

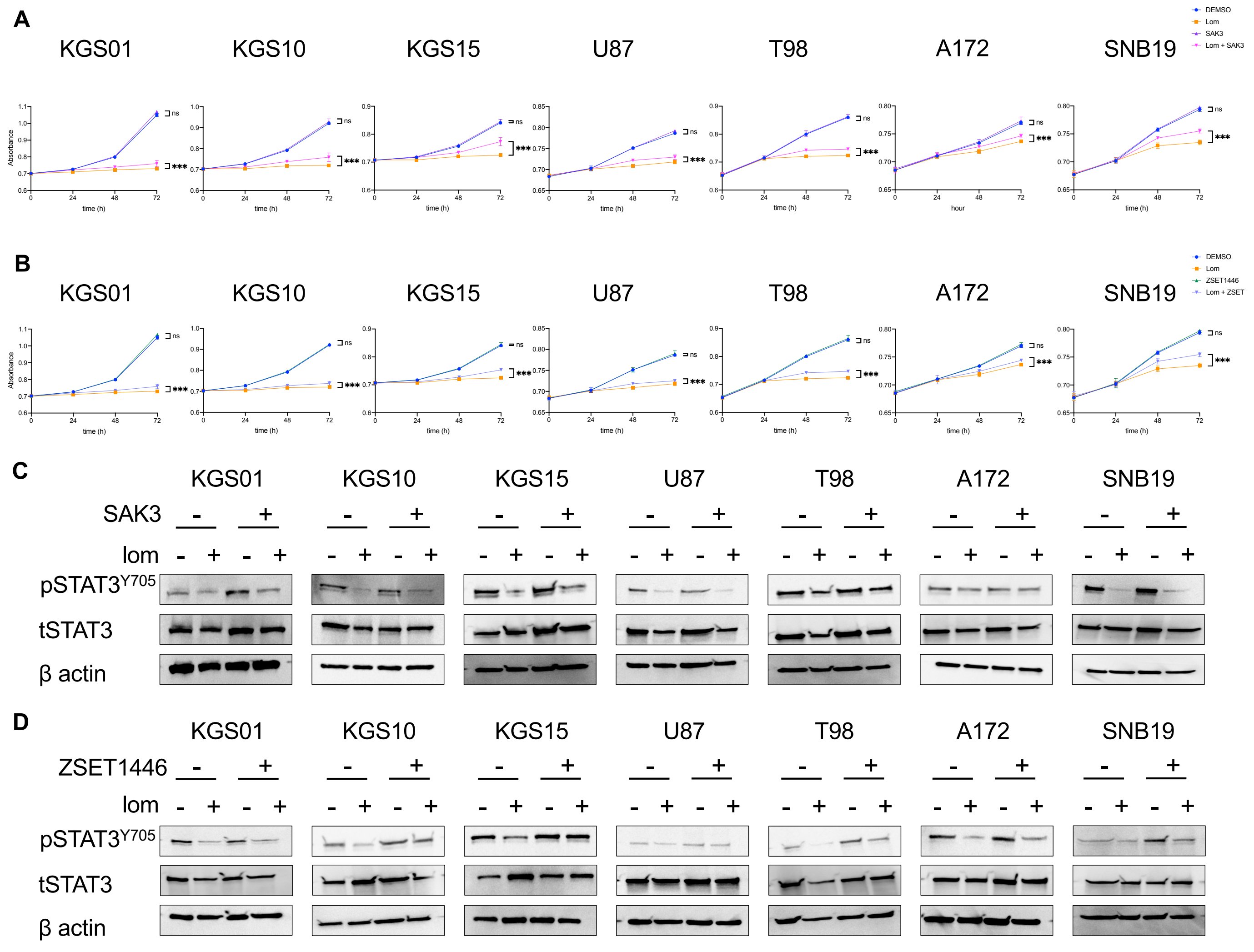

Supplementary Figure S12: Differences in properties between glioma stem/initiating cells and their differentiated cells

A: Representative images showing morphological differences between glioma stem/initiating cells (GICs; KGS01, KGS10, and KGS15) and their differentiated cells (DKGS01, DKGS10, and DKGS15). **B:** GICs were positively stained for the stem cell markers—CD133, CD44, and nestin. Tumor antispheres were differentiated into GFAP- and Olig2-positive astrocyte-like cells and Tuj1-positive neuron-like cells in DMEM supplemented with 10% FBS. **C:** Western blot analysis indicating the expression levels of SOX2, a stemness marker, in GICs and differentiated GICs.

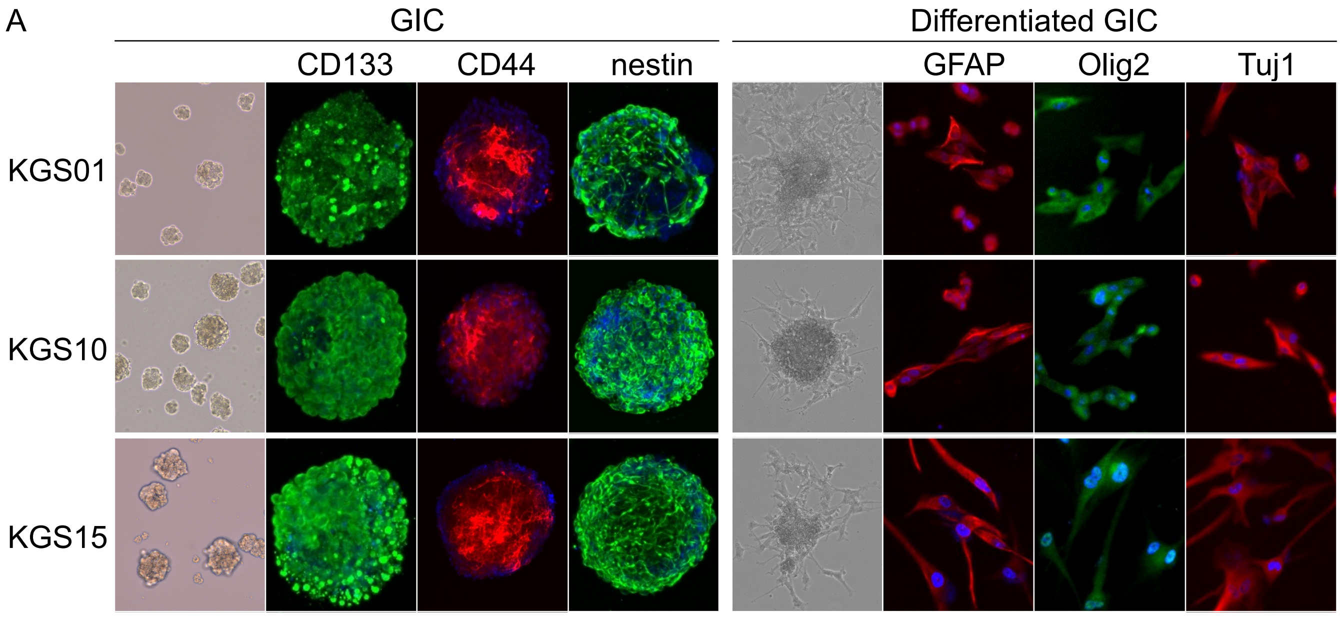

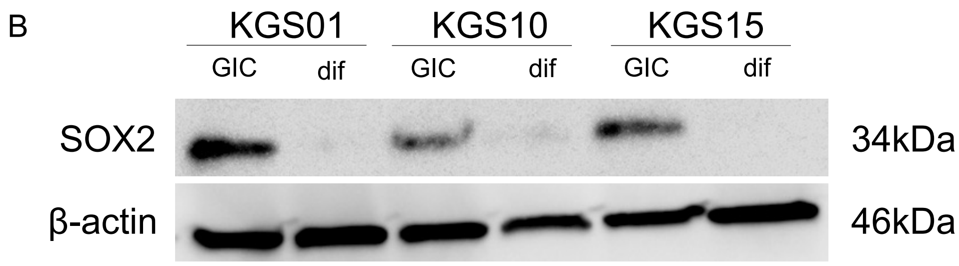

Supplementary Table 1: List of antibody types and dilutions used in this research.

Antibody	MW (kDa)	Source	Dilution	Company
Akt	60	Rabbit	1:1000 (WB)	Cell Signaling
pAkt	60	Rabbit	1:1000 (WB)	Cell Signaling
β-actin	46	Mouse	1:5000 (WB)	FUJIFILM Wako
CD133	97	Rabbit	1:400 (IF)	abcam
CD44	29-37	Mouse	1:400 (IF)	Cell Signaling
CDK4	34	Rabbit	1: 500 (WB)	Cell Signaling
CDK6	36	Rabbit	1: 500 (WB)	Cell Signaling
Cleaved PARP	89	Rabbit	1:500 (WB)	Cell Signaling
ERK	42, 44	Rabbit	1:1000 (WB)	Cell Signaling
pERK	42, 44	Rabbit	1:1000 (WB)	Cell Signaling
GFAP	55	Rabbit	1:600 (IF)	DAKO
Ki-67	359	Rabbit	1:800 (IHC)	Thermo Scientific
Nestin	260	Mouse	1:400 (IHC)<br>1:200 (IF)	BD Biosciences
Olig2	32	Rabbit	1:500 (IF)	IBL
SOX2	34	Rabbit	1:1000 (WB)	Gene Tex
STAT3	80	Rabbit	1:1000 (WB)<br>1:400 (IHC)	Cell Signaling
PARP	89	Rabbit	1:1000 (WB)	Cell Signaling
pSTAT3^{S727}	80	Rabbit	1:500 (WB)	Cell Signaling
pSTAT3^{Y705}	80	Rabbit	1:1000 (WB)<br>1:200 (IHC)	Cell Signaling
Tuj1	55	Mouse	1:200 (IF)	R&D system

Supplementary Table 2: List of Primers and Primer sequences for each gene used in this research.

Voltage-gated Ca2+-channel subunit	Primer sequence (forward; reverse)	Product (bp)
CACNA1G (T-type)	ACACTTGGAACCGGCTTGAC; AGCACACGGACTGTCCTGA	106
CACNA1H (T-type)	CTTCTTCTGCCTCGGTCAGA; TGATTACCAGCATGCTCACG	95
CACNA1I (T-type)	ATCGACTACACCCTGTGCTTCCG; GACGTAGTCGAAGAGTTTGTGGGC	162
